# Co-delivery of panobinostat and siSTAT3 using engineered M1 exosomes to establish a one-two punch therapeutic strategy for glioblastoma recurrence

**DOI:** 10.1016/j.mtbio.2025.102680

**Published:** 2025-12-13

**Authors:** Xuemeng Liu, Yaotian Hu, Yan Zhang, Chang Liu, Jingwen Wu, Ruiqi Zhao, Zhiyi Xue, Wenjing Zhou, Xiaofei Liu, Hrvoje Miletic, Yongli Gao, Chen Qiu, Jian Wang

**Affiliations:** a Department of Neurosurgery, Qilu Hospital, Cheeloo College of Medicine and Institute of Brain and Brain-Inspired Science, Shandong University, Jinan, 250012, China; b Jinan Microecological Biomedicine Shandong Laboratory and Shandong Key Laboratory of Brain Function Remodeling, Jinan, 250117, China; c Department of Oncology, Linyi People's Hospital, Xuzhou Medical University, Linyi, Shandong Province, 276000, China; d Department of Oncology, Qilu Hospital, Shandong University, Jinan, 250012, China; e Department of Blood Transfusion, Shandong Provincial Hospital Affiliated to Shandong First Medical University, Jinan, Shandong, China; f Department of Biomedicine, University of Bergen, Jonas Lies Vei 91, 5009, Bergen, Norway

**Keywords:** Glioblastoma, Exosome, M1 macrophage, Panobinostat, siSTAT3, Tumor microenvironment

## Abstract

Effectively treating recurrent glioblastoma (GB) remains a significant challenge in the clinic. Considering the multifactorial nature of GB progression, a comprehensive therapeutic strategy that directly targets both the tumor cells and its microenvironment is crucial. In this study, we developed an approach using exosomes derived from genetically modified M1 macrophages that encapsulate panobinostat and siSTAT3 to treat recurrent GB. We demonstrate that this innovative system has an innate ability to actively home to tumor cells, leveraging the inflammation-targeting capabilities of M1 macrophage-derived exosomes. These exosomes are pivotal in shifting the balance from M2 macrophages to the more favorable M1 phenotype within the tumor microenvironment. By loading the exosomes with panobinostat, a compound that faces challenges crossing the blood-brain barrier, it can efficiently access and act upon the tumor. Moreover, with the co-delivery of siSTAT3, the exosomes display various functionalities, such as inhibiting GB proliferation and invasion, preventing astrocyte reactivity, and reducing M2 macrophage infiltration. This “one-two punch” approach offers a powerful combined anticancer effect through simultaneously targeting tumor cells and reshaping the tumor microenvironment, which holds considerable promise in curbing GB recurrence and provides hope for more effective future treatments.

## Graphical abstract

**Figure:**
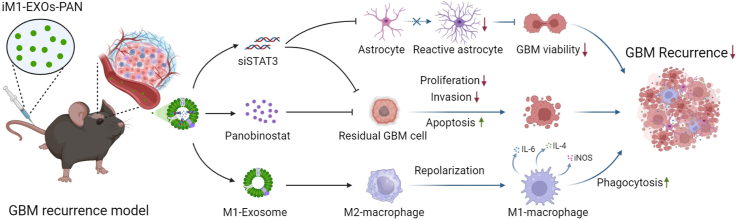

## 1 Introduction

The conventional therapies for glioblastoma (GB) have primarily focused on tumor cells or elements within the tumor microenvironment (TME). However, the diffuse and invasive growth pattern enables tumor cells to evade complete surgical resection while diminishing the efficacy of adjuvant chemotherapy and radiotherapy [1,2]. Although surgical intervention is crucial for extending survival and managing symptoms in patients, it paradoxically promotes a tumor-supportive microenvironment by amplifying inflammatory signals that contribute to GB recurrence [3,4]. Therefore, strategic therapies must evolve toward more sophisticated and targeted therapeutic approaches, particularly for recurrent GB [5].

The GB TME is highly complex, marked by persistent inflammation and cellular heterogeneity, including various cellular components such as astrocytes, endothelial cells, glioma stem-like cells, diverse immune populations, and non-cellular elements like the extracellular matrix [6]. Astrocytes, constituting the central nervous system's predominant glial cell population, maintain neural homeostasis through neurotransmitter regulation, nutritional support, and blood-brain barrier (BBB) maintenance [7,8]. In the context of GB progression, these astrocytes undergo activation, forming a reactive layer surrounding the tumor mass. This reactive astrocyte population contributes to both tissue repair mechanisms and tumor progression through the coordinated secretion of growth factors and pro-inflammatory cytokines [[9], [10], [11]]. Previous research by Okolie et al. demonstrated that surgical resection triggers widespread astrocyte activation, subsequently promoting GB recurrence through multiple mechanisms [12]. However, the mechanisms governing reactive astrocytes in GB, particularly post-resection, remain largely unexplored. Studies have shown that the signal transducer and activator of transcription 3 (STAT3) signaling is central in mediating astrocyte activation and subsequent tumor-promoting functions. Inhibitors of STAT3 signaling significantly inhibit the growth of GB cells in vitro, but have not proven effective in randomized Phase III clinical trials. One of the important reasons is that many inhibitors of STAT3 are not able to cross the blood-brain barrier (BBB) and can only act on peripheral tumors and are not able to treat brain tumors [[13], [14], [15]]. In this context, strategies targeting astrocyte reactivity inhibition, which may improve the therapeutic outcome of GB, warrant further investigation.

Another challenge in treating GB is the delivery of drugs that can sufficiently cross the BBB. Panobinostat is a potent inhibitor of class I, II, and IV histone deacetylases (HDACs), which are crucial in inhibiting STAT3 expression and have demonstrated significant potential in anti-angiogenic and antitumor applications [16]. However, a pivotal phase II clinical trial investigating the combination of panobinostat with bevacizumab in recurrent GB patients failed to demonstrate significant improvement in progression-free survival. Post-trial analysis revealed that this therapeutic limitation primarily stemmed from insufficient BBB penetration of panobinostat [17]. Thus, this limitation highlights the critical need for improved delivery systems to cross the BBB while maintaining therapeutic efficacy. Exosomes have recently emerged as promising vehicles for therapeutic delivery. They are distinguished by their natural stability, exceptional biological barrier permeability, minimal immunogenicity, and inherent cellular targeting capabilities [18]. Of particular significance is their demonstrated ability to cross the BBB more effectively than conventional delivery systems [19].

Although exosomes have been extensively studied as drug delivery vehicles, caution needs to be taken when choosing the cell of origin, as exosomes may carry undesirable or harmful cargo, such as oncogenic factors or pro-inflammatory molecules. Due to macrophages’ unique immunological properties, they have garnered attention as potential therapeutic vectors, including extended blood circulation times and specific tumor-targeting capabilities. Their intrinsic ability to respond to chemotactic signals and preferentially home to inflammatory sites makes macrophage-based delivery systems particularly promising for targeted cancer therapy [[20], [21], [22], [23]]. M1-macrophage-derived exosomes (M1-EXOs) inherit these advantageous properties from their parent cells while offering additional therapeutic benefits. Beyond their capacity to effectively deliver various anticancer agents [24], Wang PP et al. proposed that M1 macrophage exosomes can re-polarize M2 macrophages by activating the NF-κB pathway, thereby promoting the release of pro-inflammatory Th1-type cytokines to enhance macrophage-mediated tumor cell phagocytosis [25]. Choo YW, Baek S et al. also demonstrated that M1 macrophage exosomes can re-polarize M2 macrophages, though they did not elucidate the specific mechanisms involved [26,27]. In the context of GB recurrence, macrophages are a major cell population, accounting for around 50 % of stromal cells infiltrating the tumor tissue [28]. Our previous study using a GB surgical debulking model also demonstrated increased recruitment of macrophages to the tumor site [29]. Moreover, hypoxia, low nutrition, and an abundance of lactate and immunosuppressive cytokines turn tumor-infiltrating macrophages into tumor-associated macrophages (TAMs) with an M2 polarization phenotype [30]. These TAMs are capable of promoting tumor growth, angiogenesis, and tumor metastasis and are associated with a high rate of recurrence and poor prognosis in tumor patients [31]. Therefore, these M1-EXOs, when used as vehicles, may potentially have dual therapeutic effects through direct drug delivery and immune system modulation against reactive astrocytes and TAMs. These potential combined effects thus warrant further investigation, as the complex interplay between reactive astrocytes and TAMs likely contributes to the aggressive nature of recurrent GB.

Based on these findings, the present study investigated three critical aspects in association with recurrent GB and treatment: 1) The mechanistic role of STAT3 in mediating astrocyte reactivity and its subsequent effects on GB proliferation, invasion, and migration, with particular emphasis on the impact of surgical injury-induced activation; 2) The characterization and optimization of M1-EXOs' inflammatory chemotaxis and tumor-targeting capabilities, focusing on their potential as therapeutic delivery vehicles; 3) The therapeutic efficacy of engineered M1-EXOs designed to co-deliver siSTAT3 and panobinostat and their impact on tumor cells and the microenvironment.

## 2 Experimental section

### 2.1 Cell lines and cell culture

GB cell lines LN229, A172, GL261, human umbilical vein endothelial cells (HUVEC), and normal human astrocytes (NHA) were obtained from the Chinese Academy of Sciences Cell Bank (Shanghai, China), and so were the macrophage cell lines THP1 cells and Raw264.7 cells. LN229, A172, GL261, Raw264.7, and NHA cells were cultured in Dulbecco's modified Eagle's medium (DMEM, Thermo Fisher Scientific, USA) with supplementation of 10 % fetal bovine serum (FBS). THP-1 cells were cultured in 1640 medium (Gibco, USA) supplemented with 10 % FBS.

### 2.2 Transient transfection and lentiviral infection

The siRNAs targeting STAT3 (Gene Pharma, Shanghai, China) were transiently transfected into GB cells by Lipofectamine 2000 reagent (Thermo Fisher Scientific). SiRNA target sequences used in this study were as follows: siSTAT3-NC (5′-TTCTCCGAACGTGTCACGT-3′), siSTAT3#1 (5′-GCAAAGAATCACATGCCACTT-3′), siSTAT3#2 (5′-GCACAATCTACGAAGAATCAA-3′), siSTAT3#3 (5′-GGCGTCCAGTT CACTACTAAA-3′). Based on the siRNA target sequences, shRNA interference fragments targeting STAT3 were designed by OBiO-Tech (Shanghai, China).

### 2.3 Real-time qPCR (qRT-PCR)

Total RNA was extracted from Raw264.7 cells with TRIzol reagent and reverse transcribed into cDNA with the cDNA Synthesis Kit (11121ES60, YEASEN, Shanghai, China). Real-time PCR was performed on cDNA in the real-time PCR detection system (Roche, Basel, Switzerland). GAPDH served as an internal control. The sequences of the primers used for qRT-PCR are listed in Supplementary Table S1.

### 2.4 Preparation of M1-EXOs

RAW 264.7 cells were polarized to M1 macrophages by incubation with 100 ng mL^−1^ lipopolysaccharide (LPS) for 48 h, or polarized to M2 macrophages by incubation with 20 ng mL^−1^ IL‐4 for 48 h [32]. THP1 cells were first induced with phorbol-12-myristate-13-acetate (PMA) 50 ng/mL for 24 h, and then induced with 20 ng/mL IFN-γ + 100 ng/mL LPS for 24 h to obtain human-derived M1 macrophages [33]. Exosomes were purified by a differential centrifugation method, as described previously [34].

### 2.5 Preparation of M1-EXOs-panobinostat/siSTAT3 (iM1-EXOs-PAN)

The total protein content and particle concentration of M1-EXOs were measured by BCA assay and nanoparticle tracking analysis (NTA, NanoSight NS300, Malvern). First, to load the M1-EXOs with panobinostat, 100 μg of purified M1-EXOs were gently mixed with 100 μg of panobinostat. And, the mixture was sonicated (500 v, 2 kHz, 20 % power, 6 cycles by 4 s pulse/2 s pause), cooled down on ice for 2 min, and then sonicated again, as previously described [35]. Then, the unbounded panobinostat prodrugs were removed by Exosome Spin Columns (Mw 3000) (ThermoFisher, Waltham, MA, USA). Second, to load siSTAT3, ∼10^9^ exosomes were mixed with 1.5 μg siRNA (siSTAT3#3) in electroporation buffer (RNase-free PBS pH 7.4), and electroporation was applied using a single 4 mm cuvette and a Lonza Nucleofector 2 B system according to the protocol. After electroporation, the exosomes were treated with RNase to remove any siRNAs that might be bound to the exosome membrane. Finally, the electroporated exosomes were diluted with cold PBS and ultracentrifuged at 1.0 × 10^5^ g, 4 °C for 70 min to remove unbound siSTAT3 [36].

### 2.6 Characterization of M1-EXOs and iM1-EXOs-PAN

The size distribution of M1-EXOs and iM1-EXOs-PAN was analyzed by NTA. Transmission electron microscopy (TEM, JEM-2100 F, JEOL Ltd., Japan) was used to observe the morphology of M1-EXOs and iM1-EXOs-PAN. Then, M1-EXOs and iM1-EXOs-PAN were characterized by western blotting using antibodies against CD81 (66,866–1, Proteintech, Wuhan, China), CD9 (13403 S, CST), Calnexin (2679 T, CST) and TSG101 (ab125011, Abcam). Fluorescence co-localization of PKH67-labeled exosomes with Cy3-labeled siSTAT3 was recorded by confocal laser scanning microscopy (CLSM). To measure the amount of siSTAT3 loaded into exosomes, Cy3-labeled siSTAT3 was used, and fluorescent signal intensity was evaluated by a microplate reader (*Synerg*y2, Bio-Tek, USA) and calculated based on a pre-established standard curve.

### 2.7 High-performance liquid chromatography (HPLC) assay

The concentration of non-loaded panobinostat in the supernatant was evaluated by HPLC (Agilent, USA). We performed the analysis using a Welch Ultimate Plus C18 column (250 × 4.6 mm, 5 μm). A DAD detector was used with a detection wavelength of 280 nm. The flow rate was 1 mL/min, column temperature was 31 °C, and injection volume was 1 μL. Mobile phase A was 0.1 % trifluoroacetic acid in water, mobile phase B was acetonitrile, and gradient elution was employed. Besides, the standard solution is 10 mg/mL. We diluted it with methanol to a 1 mg/mL solution, run it on the instrument, and recorded the chromatogram. The chromatogram acquisition and integration of panobinostat were processed using Chemstation software.

### 2.8 Cellular uptake assay

Exosomes were labeled with PKH67 (MINI67, Sigma). NHA and GB cells were seeded at a density of 2.0 × 10^4^ cells into a confocal dish and then incubated with PKH67-labeled exosomes for 12 h. After incubation, cells were collected to stain nuclei with DAPI (P0131, Beyotime, Shanghai, China) and cytoskeleton with TRITC Phalloidin (40734ES75, YEASEN, Shanghai, China) for CLSM analysis.

### 2.9 Effects of inflammatory stimuli on iM1-EXOs-PAN chemotaxis in vitro

GB cells (2.0 × 10^4^) were seeded onto the bottom chamber of a 24-well trans-well, whereas human umbilical vein endothelial cells (HUVEC) were seeded on a Matrigel pre-coated upper chamber (1.0 × 10^5^ cells/well). Monolayered HUVEC with TEER values greater than 300 Ω cm^2^ were adopted as the BBB model. Then, 0.2 μg/μL of PKH67-labeled iM1-EXOs-PAN was added to the upper chamber, and FBS-free medium with or without fMLP (100 μΜ, HY-P0224, MCE) was added to the lower chamber [37]. After 4, 8, and 12 h of incubation, the cellular uptake of iM1-EXOs-PAN in GB cells and HUVEC was observed by CLSM.

### 2.10 Cell viability assay

GB cells were co-cultured with NHAs transfected with different shRNAs. Their proliferation was evaluated with CellTrace™ CFSE Cell Proliferation Kit (C34554, Thermo Fisher Scientific, USA). GB cell proliferation after different treatments was also assessed with the Cell Counting Kit (CCK-8, YEASEN, Shanghai, China). The absorbance was tested at 450 nm with the EnSight Multimode Plate Reader (PerkinElmer, Singapore).

### 2.11 Colony formation assay

GB cells (800 cells/well) were seeded into 6-well plates, which contained 2 mL of complete medium. Transient transfections were conducted in 6-well plates with different exosomes or PBS per well. After 2 weeks of incubation in a cell incubator, colonies were fixed with 4 % paraformaldehyde for 15 min and stained with 0.5 % crystal violet for 20 min. Then, colonies (>50 cells) were counted with a bright field microscopy.

### 2.12 In vitro mechanical scratch injury

In vitro surgery scratch injury was performed using a well-established model of mechanical scratch injury. To exploit the effects of STAT3 on mechanical scratch injury in vitro, NHA cells subjected to mechanical scratch injury were scratched using a 200-μL yellow gunshot with a scratch width of 1 mm, as previously described [38,39]. NHA cell line was transfected with shNC or shSTAT3 before subjected to mechanical scratch injury.

### 2.13 Protein extraction and protein immunoblotting

The cells were treated for 48 h, digested with trypsin, centrifuged at 1000 rpm for 3 min, and lysed for 15 min in RIPA lysis buffer (P0013B, Beyotime, Shanghai, China). The lysate was centrifuged, and the supernatant protein concentration was measured by the BCA assay kit (P0009, Beyotime, Shanghai, China). Protein lysates (25 μg) were separated with 10 % sodium dodecyl sulfate-polyacrylamide gel electrophoresis and transferred to PVDF membranes (GVW2932A, 0.22 μm, Millipore Sigma; Burlington, MA, USA). The PVDF membranes were blocked in 5 % skim milk for 1 h and incubated with primary antibodies overnight at 4 °C. After incubated with secondary antibodies (1:5000) at room temperature for 1 h, membranes were exposed to ECL reagent and visualized on the Chemiluminescence Imager (Bio-Rad; Hercules, CA, USA).

### 2.14 Trans-well invasion assay

Cells (2 × 10^4^) in 200 μL DMEM medium without FBS were seeded into the upper chamber, and a 600 μL complete medium containing 30 % FBS was added to the lower chamber. After 36 h, the cells were fixed with 4 % formalin for 20 min and stained with 0.5 % crystal violet for 20 min. After three washes with PBS, three random areas in the images obtained under light microscopy were used to calculate the migrated cells.

### 2.15 GB migration in vitro

GB cells (LN229&A172) and NHA-shNC/NHA-shSTAT3 were seeded separately into adjacent wells located 0.5 mm apart in 2-chamber culture inserts (Eubio 80,241, Ibidi, Martinsried, Germany), placed in 24-well plates, and incubated overnight in complete media. Culture inserts were removed, and cells were imaged over 24 h for migration.

### 2.16 Live & dead cell cytotoxicity assay

First, we prepared the working solution (2 μM calcein AM, 8 μM PI). 5 μL of 4 mM calcein AM reservoir (reagent A, KGAF001, Keygen Biotech, Jiangsu, China) and 5 μL of 16 mM PI storage solution (reagent B, KGAF001, Keygen Biotech, Jiangsu, China) were added to 10 mL of PBS. Cells were prepared with certain treatments in 24-well plates. 300 μL working solution incubated the monolayer cells in each well at room temperature for 45 min. Then, the staining working solution was aspirated, and 300 μL PBS covered the cells. Labeled cells were observed under a fluorescence microscope.

### 2.17 EdU apollo cell proliferation assay

GB cells (20,000/well) were seeded into 24-well plates. Certain treatments were carried out according to the experimental needs. 300 μL of 50 μM EdU medium (reagent A: complete medium = 1:1000) was adde to each well for 2 h and discard the medium. After fixation for 30 min, 300 μL of 2 mg/mL of glycine was added, and after 5 min of decolorization, was discarded. 300 μL of 0.5 % Tritonx-100 incubated cells in each well for 10 min, enhancing cell membrane permeability. 250 μL of 1 × Apollo staining solution was added to the cells and the staining solution was discarded after 30 min of dark incubation at room temperature. 200 μL 1 × Hoechst 33,342 reaction solution (reagent F diluted with deionized water at a ratio of 1:100) incubated the cells for 30 min at room temperature and away from light. The images were captured by fluorescent microscope. The percentage of EdU^+^ cells was counted to judge the cell proliferation.

### 2.18 Annexin V-FITC/PI assay for cell apoptosis

5 × 10^5^ cells were resuspended by adding 100 μL 1 × Binding Buffer (40302-C, YEASEN, Shanghai, China). 5 μL Annexin V-FITC (40302-A, YEASEN, Shanghai, China) and 10 μL PI staining solution (40302-B, YEASEN, Shanghai, China) were added and mixed gently. The samples were kept in the dark at room temperature for 15 min and examined by flow cytometry within 1 h.

### 2.19 Living cells Caspase-3 activity and annexin V cell apoptosis assay

The cells were cultured in 24-well. After apoptosis induction, the cell culture medium was aspirated, and the cells were washed once by PBS. 194 μL Annexin V-mCherry Binding Buffer (C1077M − 3, Beyotime, Shanghai, China), 5 μL Annexin V-mCherry (C1077M − 1, Beyotime, Shanghai, China) and 1 μL GreenNuc™ Caspase-3 Substrate (C1077M − 2, Beyotime, Shanghai, China) were mixed and added in the well. The sample was incubated in the dark at room temperature for 30 min and placed in ice after incubation. The GreenNuc™-DNA showed green fluorescence (Ex/Em = 500 nm/530 nm), and Annexin V-mCherry showed red fluorescence (Ex/Em = 587 nm/610 nm) under a fluorescence microscope.

### 2.20 Immunofluorescence (IF) staining

After deparaffinization and antigen retrieval treatment, sections were blocked for 30 min with bovine serum and then incubated for 2 h at room temperature or overnight at 4 °C with anti-glial fibrillary acidic protein (GFAP, 12389 S, CST), CD86 (ab239075, Abcam) and CD206 (24595 S, CST) antibodies. The samples were treated for 45 min at room temperature with secondary antibodies, which were fluorescently species-appropriate, at a dilution of 1:500. DAPI (P0131, Beyotime, Shanghai, China) was used to stain nuclei. To acquire the results, the sections were scanned with CLSM.

### 2.21 Immunohistochemistry (IHC) staining

Sections of paraffin-embedded tumors/tissue were dewaxed and hydrated, and antigen retrieval was performed in 1 × EDTA. Then, the staining was performed using IHC kit (36312ES50, YEASEN, Shanghai, China). The sections were blocked with serum for 30 min, incubated with the primary antibodies Ki67 (ab15580, Abcam), Bax (ab32503, Abcam) and STAT3 (ab68153, Abcam) diluted in PBS (1:200) overnight at 4 °C and finally incubated with secondary antibody at room temperature for 1 h. For detection, 100 μL of DAB staining solution was added to each sample and images were acquired with a digital panoramic scanner (WISLEAP, Jiangsu, China).

### 2.22 M2 macrophages induction and repolarization

Raw264.7 macrophages were induced into the M2 phenotype by 20 ng mL^−1^ IL‐4 for 48 h. Then, certain exosomes, LPS (100 ng mL^−1^) or PBS were individually treated for the M2 macrophages at 37 °C for 24 h. To observe the degree of repolarization, the protein expressions of CD86 (M1 macrophage marker) and CD206 (M2 macrophage marker) were evaluated by immunofluorescent staining. mRNA expression of IL-6, TNF-α, iNOS and IL-4 were detected by qRT-PCR. The in vitro phagocytosis capability assay is as previously reported [40].

### 2.23 TUNEL assay

The One Step TUNEL Apoptosis Assay Kit (C1088, Beyotime, Shanghai, China) was used to evaluate apoptosis in sections from tissues exposed to different treatments. Tumor sections were dewaxed, hydrated and treated with 20 μg/mL proteinase K for 30 min at 37 °C. The TUNEL assay was performed according to the manufacturer's instructions.

### 2.24 Construction of GB recurrent animal models

The animal study procedures were approved by the Scientific Research Ethics Committee of Qilu Hospital, Shandong University (approval No. DWLL-2021-127, Shandong, China). The study was conducted in full compliance with relevant regulations and guidelines. An intracranial GB mouse model was established through stereotactic inoculation of GL261^luci^ cells (150,000 cells per mouse in 5 μL of PBS) into the brain (2 mm lateral and 0.5 mm anterior to the bregma; 2 mm of depth) of the mice that were anesthetized using inhalational 1–5 % isoflurane mixed with oxygen. As described above, the GB mouse model was first established to create the post-surgery GB mouse model. On day 8 after inoculation, a small circular portion of the skull (∼4 mm in diameter) was removed from each mouse using a high-speed cranial drill, and the tumor-bearing mice underwent surgical resection of GB using ultrasonic suction instruments.

### 2.25 Brain tumor-targetability and anti-tumor effect of iM1-EXOs-PAN

The brain-tumor targetability and therapeutic efficacy of iM1-EXOs-PAN were evaluated on a mouse GB surgical resection model implanted with GL261 cells. We first monitored the in vivo biodistribution of DiR-labeled iM1-EXOs-PAN after intravenous administration in different mouse models (normal mice, GL261-bearing mice, surgically treated GL261-bearing mice). At 2, 4, 8, 12, and 24 h, the biodistribution of DiR-labeled iM1-EXOs-PAN (90 μg) fluorescence in each group was monitored by an in vivo spectrum imaging system (IVIS-200, Xenogen, Alameda, CA, USA). To study the antitumor efficacy in vivo, 40 post-operative male C57 mice were randomly divided into five groups (n = 8) based on the bioluminescence signal of residual tumors in mice detected by the IVIS spectrum on the first day after surgery (1 day). Mice were intravenously injected with PBS, M1-EXOs, iM1-EXOs, M1-EXOs-PAN, and iM1-EXOs-PAN (with an equivalent panobinostat concentration of 0.5 mg/kg) every 3 days for 5 times. Bioluminescence signals of tumors were monitored by the IVIS spectrum. Survival (in days) was determined as the number of days starting from implantation (day 1) to death. Mice were euthanized at the end of the experiment. Part of the excited brain tissues was digested into single cells in which CD86 and CD206 were marked for flowcytometric analysis. The rest was snap frozen in liquid nitrogen or formalin-fixed for further analysis (TUNEL assay, IF and IHC staining).

### 2.26 In vitro bioluminescence assay

Luciferase-labeled GL261 cells (20,000 cells per well) were seeded into white opaque 96-well plates and co-cultured with pre-treated macrophages. After 24 h, the supernatant was removed, and D-luciferin substrate working solution (103,404-75-7, YEASEN, Shanghai, China) was added, followed by incubation at 37 °C for 5 min. Luminescence was then measured using a full-wavelength microplate reader.

### 2.27 Plotting and statistical analysis

Every experiment was conducted independently three times. Statistical analysis of the data was conducted using GraphPad Prism 8 software. Data are presented as the means ± standard deviation (S.D.). Two‐tailed Student's *t*‐test for direct comparisons and analysis of variance (ANOVA) for multigroup comparisons were performed to calculate *P* values for different experimental purposes. Statistical analysis was taken to differ for the following *P* values: *∗P* < 0.05, *∗∗P* < 0.01, *∗∗∗P* < 0.001 and n. s = not significant.

## 3 Results

### 3.1 STAT3 regulates GB cell aggressive behavior

We first analyzed the TCGA and CGGA databases, which revealed elevated STAT3 expression in GB, with expression levels correlating positively with glioma grade (Fig. S1A–B). Overall survival (OS) analysis demonstrated that patients with high STAT3 expression had significantly shorter life expectancy than those with low expression, based on CGGA data (Fig. S1C). STAT3 emerged as a decisive risk factor for survival (hazard ratio 1.1, confidence interval 1.03–1.1, *P* < 0.001), indicating its potential as a prognostic marker for GB patients (Fig. S1D). Then, we performed a Time-dependent ROC curve analysis, which yielded areas under the curves (AUCs) of 0.769, 0.741, and 0.688 for 1-, 3-, and 5-year survival, respectively (Fig. S1E). KEGG pathway analysis of the TCGA data revealed that STAT3-associated genes were enriched in critical regulatory pathways, including JAK-STAT, focal adhesion, notch signaling, apoptosis, and P53 signaling, all of which significantly influence GB's malignant behavior (Fig. S1F).

Additionally, STAT3 expression was notably higher in recurrent GB patients compared to primary cases (Fig. S2A). IHC analysis demonstrated increased STAT3-positive cells in higher-grade glioma tissues, with the highest levels observed in recurrent GB tissues (Fig. S2B).

To experimentally validate STAT3's functional role in GB growth, we conducted knockdown experiments using three different STAT3 siRNA in normal human astrocytes (NHA) and GB cell lines LN229, and A172. Transfection of si-STAT3#S1, #S2, #S3, revealed efficient knockdown compared to an empty vector control (siNC), which was confirmed by Western blot (Fig. S3A). For subsequent experiments, we chose si-STAT3#S2 and si-STAT3#S3 due to the most efficient knockdown. CCK8 and EdU assays demonstrated that STAT3 knockdown significantly inhibits the proliferation of GB cell lines LN229 and A172 (Fig. S3B–C). Transwell assays showed that STAT3 knockdown significantly reduced cell migration capacity (Fig. S3D). STAT3 depletion induced GB cell apoptosis as shown by Annexin/PI flow cytometry analysis (Fig. S3E–F). These data were further confirmed by downregulation of BCL2, N-cadherin and Vimentin, and concurrent upregulation of BAX (Fig. S3G).

### 3.2 STAT3 Mediates a Promotion Effect of Activated Astrocytes on GB cells

**Fig. 1:**
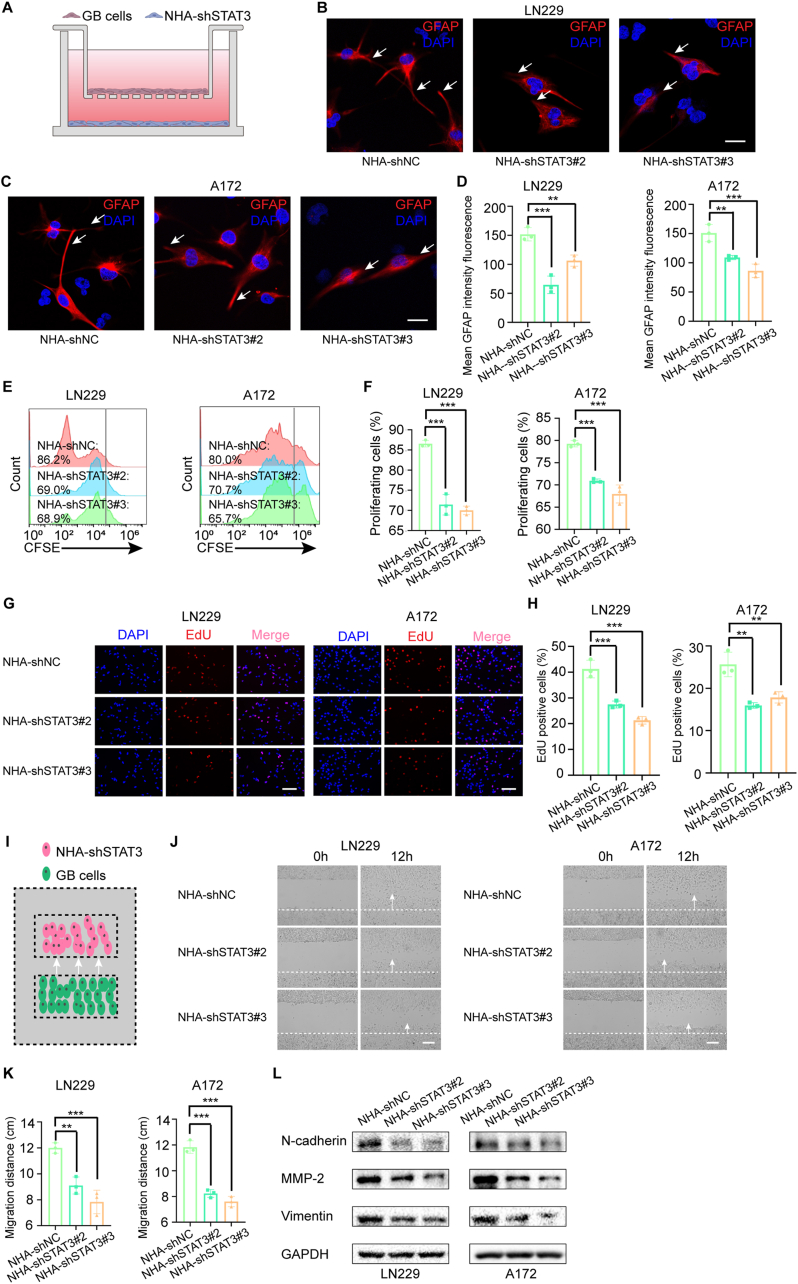
STAT3 Mediates a Promotion Effect of Activated Astrocytes on GB Cells. (A) Schematic representation of the co-culture of GB cells and NHAs transfected with shSTAT3 (NHA-shSTAT3). The transwell co-culture system containing GB cells (LN229 or A172) in the upper chamber and NHA-shSTAT3 cells in the bottom chamber. (B–C) Representative immunofluorescence images of GFAP (Red) in NHAs (shNC, shSTAT3#2, or shSTAT3#3) co-cultured with GB cells (LN229 or A172). White arrows show the morphological changes towards a multipolar star shape or a classical fibroblastic form. Scale bar, 20 μm. (D) Bar plot represents the mean fluorescence intensity of GFAP expression in NHAs (shNC, shSTAT3#2, or shSTAT3#3) co-cultured with GB cells. (*E*–F) CFSE-labeled LN229 and A172 were co-cultured with STAT3-depleted NHAs (shNC, shSTAT3#2 or shSTAT3#3). The proliferation of LN229 and A172 was evaluated by CFSE dilution using flow cytometry after 48 h of co-culture. (G–H) EdU assay of LN229 and A172 cells co-cultured with STAT3-depleted NHAs as indicated. Scale bar, 50 μm. Data points in histograms are the percent of EdU positive cells (blue: all cells; red: proliferating cells). (I) Schematic representation for the 2-chamber culture inserts migration assay of the co-cultured GB cells and NHAs transfected with shRNAs (shNC, shSTAT3#2, or shSTAT3#3). GB cells (LN229 or A172) and NHA (shNC, shSTAT3#2 or shSTAT3#3) were seeded separately into adjacent wells located 0.5 mm apart in 2-chamber culture inserts (Ibidi), placed in 24-well plates, and incubated overnight in complete media. Culture inserts were removed and the cells were imaged over 24 h for migration. (J–K) Representative images and statistical analysis diagrams of migration of LN229 and A172 cells towards STAT3-depleted NHA cells. White arrows show the direction of GB cell migration. Scale bar, 50 μm. (L) Western-blot analysis of N-cadherin, MMP-2, Vimentin and GAPDH expression in A172 and LN229 cells cocultured with STAT3-depleted NHAs as indicated. Data are presented as the means ± S.D. (n = 3). One-way ANOVA was used for multigroup comparisons. ∗∗*P* < 0.01 and ∗∗∗*P* < 0.001. (For interpretation of the references to color in this figure legend, the reader is referred to the Web version of this article.)

GB cells have the ability to activate astrocytes, and these activated astrocytes have recently been described to stimulate tumor cell proliferation, invasion, and resistance to therapies [41]. Interestingly, STAT3 activation is necessary for the reactive transformation of perineuronal astrocytes [42,43]. However, the role of STAT3 in the crosstalk between astrocytes and GB cells remains uninvestigated. Here, we knocked down the expression of STAT3 in NHA with two independent short hairpin RNAs (shRNA). Cells were infected with lentiviruses expressing sh-STAT3#S2 or #S3, or the empty vector control (shNC). Then, we used a trans-well cell co-culture model at ratio 2:1 GB: NHA-shSTAT3 to investigate STAT3's role in astrocyte activation in response to GB cells (Fig. 1A). Astrocyte reactivity is characterized by morphological changes with enhancement of cytoplasmic processes and upregulation of GFAP, a marker of astrocyte activation. In reactive gliosis, astrocytes typically upregulate GFAP expression in response to brain injury, stroke, and cancer [11]. We observed that astrocyte activation by LN229 and A172 cells was significantly attenuated in shSTAT3#2 or shSTAT3#3 NHA cells. The activated astrocytes in the NHA-shNC group exhibited morphological changes towards a multipolar star shape (white arrows) compared to astrocytes in NHA-shSTAT3#2 and NHA-shSTAT3#3 groups, which showed a classical fibroblastic form (white arrows). Furthermore, the GFAP fluorescence intensity was significantly decreased in NHA-shSTAT3#2 and NHA-shSTAT3#3 groups (Fig. 1B–D). In co-culture experiments, the CFSE intensity of LN229 and A172 GB cells co-cultured with shSTAT3#2 or shSTAT3#3 NHA cells was significantly higher compared to that of control groups. This indicates that shSTAT3#2 or shSTAT3#3 NHA cells reduced the frequency of cell division of co-cultured LN229 and A172 GB cells (Fig. 1E and F). Consistent with these findings, EdU analysis demonstrated that LN229 and A172 GB cells exhibited reduced DNA replication when co-cultured with shSTAT3#2 or shSTAT3#3 NHA cells (Fig. 1G and H). Collectively, these co-culture experiments revealed that shSTAT3 NHA cells significantly attenuated the proliferation-promoting effects on GB cells compared to control NHA cells. Next, we examined the effects of astrocyte on GB cell migration and proliferation using a 2-chamber coculture system (Fig. 1I). In contrast to the effects of NHA-shNC, both NHA-shSTAT3#2 and NHA-shSTAT3#3 induced a significant decrease in migration of cocultured GB cells over 24 h (Fig. 1J and K). Consistent with the functional assays, Western blot analysis demonstrated that STAT3-knockdown NHAs markedly reduced the protein levels of N-cadherin, MMP2, and Vimentin in co-cultured GB cells, confirming their inhibitory role in GB cell migration (Fig. 1L). We further investigated the role of STAT3-mediated activation of NHAs in promoting GB growth in vivo. LN229^luci^ cells and NHAs (shNC, shSTAT3#2 or shSTAT3#3) were co-implanted into the brains of nude mice. The tumor volume measured by bioluminescence imaging at 7, 14, and 21 day post implantation showed that the size of the tumors in the LN229 co-implanted NHA-shNC group was significantly larger than that in mice in the other two groups (Fig. S4A–B). The astrocytic marker GFAP was also stained. Compared to the other two groups, the LN229 co-implanted NHA-shNC group exhibited more atypical NHA cells with elevated GFAP expression and a marked increase in branching (Fig. S4C).

### 3.3 STAT3 is essential for activation of astrocytes due to mechanical injury

**Fig. 2:**
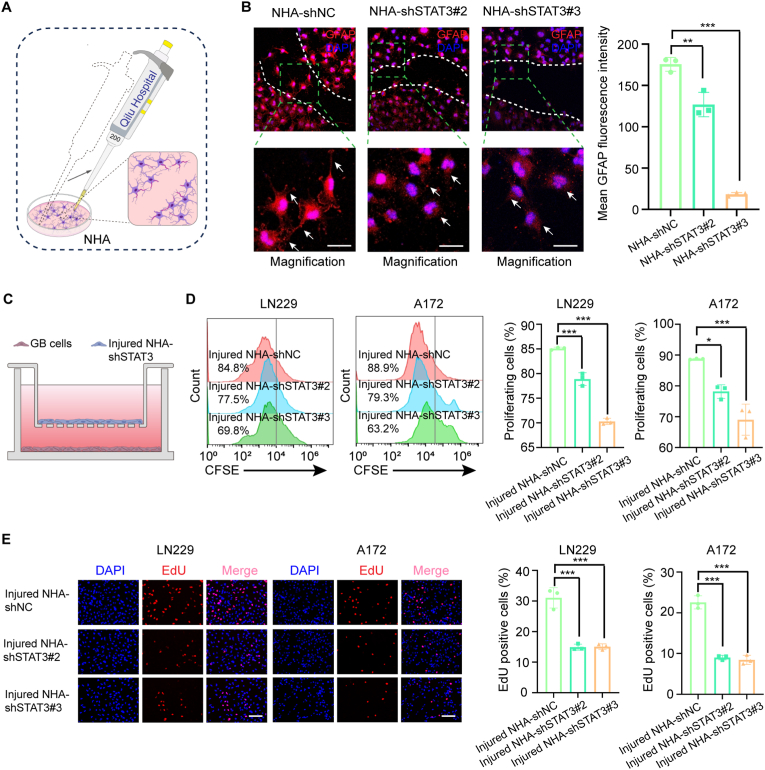
STAT3 regulates the activation of injured astrocytes by scratch. (A) A schematic illustration to mimick surgical injuries with scratches. NHA cells (shNC, shSTAT3#2 or shSTAT3#3) were seeded in dishes and scratched by a 200-μl yellow pipette tip. (B) Representative immunofluorescence images of GFAP (Red) in injured STAT3-depleted NHAs by scratch as indicated. White arrows show the morphological changes towards a multipolar star shape or a classical fibroblastic form. Bar plot represents the mean fluorescence intensity of GFAP expression in injured astrocytes with or without STAT3 depletion. Scale bar, 50 μm. (C) Schematic representation for the co-culture assay of GB cells and injured STAT3-depleted NHAs. The transwell co-culture system containing NHA cells (injured NHA-shNC, injured NHA-shSTAT3#2 or injured NHA-shSTAT3#3) in the upper chamber and GB cells in the bottom chamber. (D) CFSE-labeled LN229 or A172 were co-cultured with injured STAT3-depleted NHAs (shNC, shSTAT3#2 or shSTAT3#3). The proliferation of LN229 and A172 cells were evaluated by CFSE expression using flow cytometry after 48 h of co-culture. (E) EdU assay of GB cells co-cultured with injured STAT3-depleted NHAs. Scale bar, 50 μm. Data are presented as the means ± S.D. (n = 3). One-way ANOVA was used for multigroup comparisons. ∗*P* < 0.05, ∗∗*P* < 0.01 and ∗∗∗*P* < 0.001. (For interpretation of the references to color in this figure legend, the reader is referred to the Web version of this article.)

Surgical resection is a standard treatment for GB; however, very little is known about how the tumor microenvironment changes during this procedure. The study by Okolie et al. showed that astrocyte activation following surgical resection induces transcriptome and secretome alterations that promote tumor proliferation and migration [12]. Thus, we investigated whether STAT3 is involved in the activation response of NHA cells to mechanical injury. We first inflicted a scratch injury on cultured astrocytes using a pipette tip, as an in vitro model mimicking the trauma induced by surgical resection, as shown in Fig. 2A [38,39]. STAT3 knockdown in NHA significantly suppressed injury-induced astrocyte activation, as demonstrated by significant downregulation of mean GFAP fluorescence intensity (Fig. 2B). In a cell co-culture model at a ratio 2:1 GB: injured NHA-shSTAT3 (Fig. 2C), flow cytometry assays revealed that injured NHA-shSTAT3 cells exhibited significantly reduced proliferation-promoting effects on GB cells compared to injured NHA-shNC controls (Fig. 2D). This was further confirmed by EdU experiments (Fig. 2E).

### 3.4 Development and characterization of iM1-EXOs-PAN nanoformulation

**Fig. 3:**
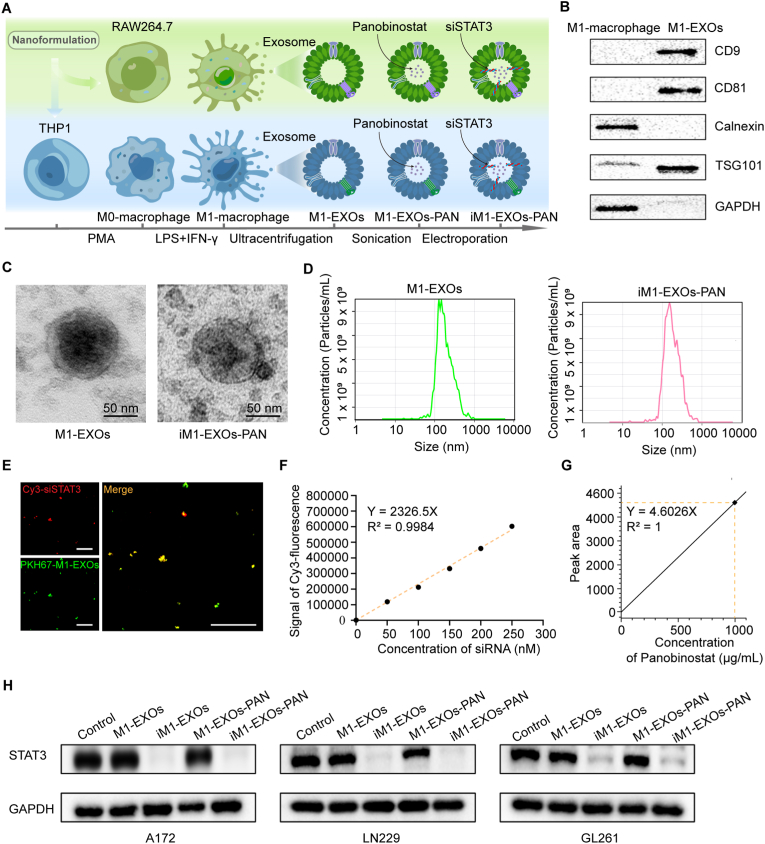
Construction of iM1-EXOs-PAN nanoformulation. (A) Scheme depicting the acquisition process of M1-EXOs and the preparation of a siSTAT3 and panobinostat co-delivery system based on M1-EXOs. THP1 and RAW264.7 cells were induced into M1 macrophages using PMA, LPS, and IFN-γ, and their exosomes were extracted via ultracentrifugation. Subsequently, panobinostat and siSTAT3 were encapsulated into the exosomes using ultrasonication and electroporation, respectively, yielding the final nanoformulation iM1-EXOs-PAN. (B) Western-blot analysis of exosomal protein biomarkers (CD9, CD81 and TSG101). (C) Representative TEM images of M1-EXOs and iM1-EXOs-PAN. Scale bar, 50 nm. (D) NTA analysis of M1-EXOs and iM1-EXOs-PAN. (E) Confocal imaging and co-localization analyses of PKH67-M1-EXOs (green) and Cy3-siSTAT3 (red). Scale bar, 10 μm. (F) The concentration standard curve of Cy3-siSTAT3 was measured by the fluorescent intensity of iM1-EXOs-PAN. (G) The standard concentration curve of panobinostat was measured by HPLC. (H) Western blot analysis of STAT3 protein levels in A172, LN229 and GL261 cells after treatment with different nanoformulations. (For interpretation of the references to color in this figure legend, the reader is referred to the Web version of this article.)

Having demonstrated that inhibition of STAT3 not only directly inhibits tumor growth and invasion but also reduces astrocytes' ability to promote tumor growth, this treatment strategy is highly attractive for targeting both tumor cells and their microenvironment. Thus, we evaluated exosomes derived from M1 macrophages (M1-EXOs) as potential carriers for co-delivering panobinostat and siSTAT3 to block a dual-target effectively. M1 macrophages were generated through cytokine stimulation of murine RAW264.7 and human THP-1 cell lines. RAW264.7 cells were directly polarized to M1 macrophages using LPS [32]. For THP-1 cells, differentiation was achieved in two steps: first to M0 macrophages with PMA for 24 h, followed by M1 polarization using the same LPS/IFN-γ cocktail [33]. The preparation protocol for this dual-delivery system is outlined in Fig. 3A. Western blot analysis confirmed that M1-EXOs expressed characteristic exosomal protein biomarkers TSG101, CD9, and CD81 (Fig. 3B). TEM revealed that loading of panobinostat and siSTAT3 did not alter the characteristic round, double-membrane structure of the exosomes (Fig. 3C). NTA showed consistent size distribution around 110 nm before and after drug loading (Fig. 3D), indicating preservation of essential physicochemical properties. Fluorescence microscopy demonstrated successful siRNA loading, evidenced by co-localization of PKH67-M1-EXOs (green fluorescence) with Cy3-siSTAT3 (red fluorescence) (Fig. 3E). The siRNA loading capacity was quantified at 0.27 ± 0.02 μg per 10^10^ M1-EXOs (Fig. 3F). The mass spectrometry peak diagram of panobinostat in the HPLC assay is provided as Fig. S5 (Fig. S5). And, the encapsulation efficiency and loading capacity of panobinostat in M1-EXOs-PAN determined by HPLC analysis were 18.9 % and 6.51 %, respectively (Fig. 3G). To confirm an efficient intracellular delivery, we studied the changes of STAT3 in GB cells with different treatments. The Western blot results showed obvious decrease of the STAT3 protein levels in A172, LN229 and GL261 cells treated with iM1-EXOs and iM1-EXOs-PAN (Fig. 3H). Taken together, the M1-EXOs can encapsulate both siSTAT3 and panobinostat without changing exosomes' physicochemical characteristics or structure.

### 3.5 BBB penetration and inflammatory chemotaxis of iM1-EXOs-PAN

**Fig. 4:**
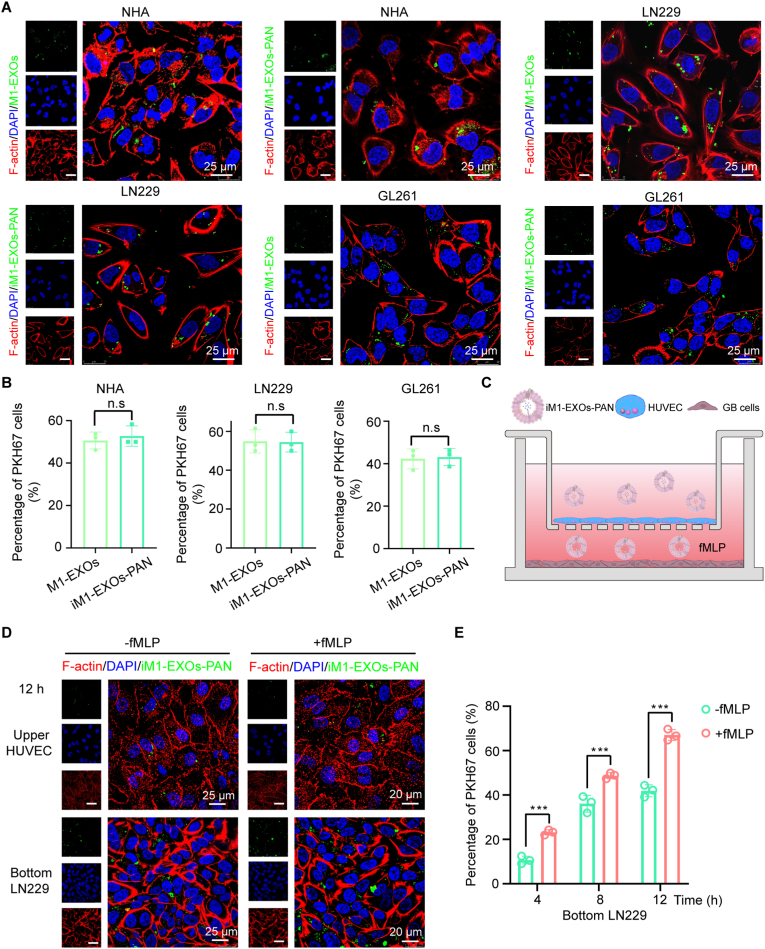
Inflammation-directed targeted delivery in vitro. (A) Representative confocal images of the uptake of M1-EXOs and iM1-EXOs-PAN by GB cells. Scale bar, 25 μm. (B) Quantification of the PKH67‐positive cell ratio based on the confocal microscopy images. (C) Schematic illustration of the in vitro BBB model using a trans-well system to evaluate the penetration capability of iM1-EXOs-PAN across the endothelial monolayer. The transwell co-culture system containing HUVEC cells (simulating the vascular endothelial cells of BBB) in the upper chamber and GB cells in the bottom chamber. And, iM1-EXOs-PAN was added to the upper chamber, and FBS free medium with or without fMLP (100 μM, MCE, HY-P0224) was added to the lower chamber. (D) Representative images of HUVECs and LN229 cells up-taking the nanoformulation treated with chemotactic peptide (+fMLP, 100 μM) or none (-fMLP) at 12 h. Scale bar, 20 μm. (E) The percentage of PKH67 positive cells treated with chemotactic peptide (+fMLP, 100 μM) or none (-fMLP) at 4, 8, and 12 h. Statistical significance was calculated using two-way ANOVA. n. s = not significant, and ∗∗∗*P* < 0.001.

Cellular uptake of exosomes is a prerequisite for drug action, which we tested in our experimental system. After incubating PKH67 (green fluorescence) labeled M1 exosomes with NHA or GB cells for 12 h in vitro, immunofluorescence imaging demonstrated successful uptake of M1-EXOs by NHA, LN229, A172, and GL261 cells. We then further investigated whether M1 exosomes loaded with panobinostat and siSTAT3 could be taken up by these cells. The immunofluorescence results demonstrated that the loading of panobinostat and siSTAT3 into exosomes did not affect their uptake (Fig. 4A and B, S6A). To evaluate BBB penetration and inflammation-driven properties, we employed an in vitro BBB model using HUVEC cells (Fig. 4C). The HUVEC monolayer exhibited characteristic “paving stone" morphology with TEER values exceeding 300 Ω cm^2^ (Fig. 4D, S6B). Time-course analysis revealed progressive accumulation of iM1-EXOs-PAN/PKH67 in GB cells in the bottom chamber at 4, 8, and 12 h post-administration. Notably, fMLP stimulation significantly enhanced iM1-EXOs-PAN/PKH67 penetration through the BBB model. The concurrent decrease in HUVEC-associated fluorescence under fMLP stimulation suggested active exosome trafficking toward inflammatory sites, demonstrating the chemotactic capabilities inherited from macrophages, which can efficiently respond to inflammation and penetrate the BBB in vitro (Fig. 4D and E, S6B).

### 3.6 Combined chemo-gene therapy effects of iM1-EXOs-PAN in vitro

**Fig. 5:**
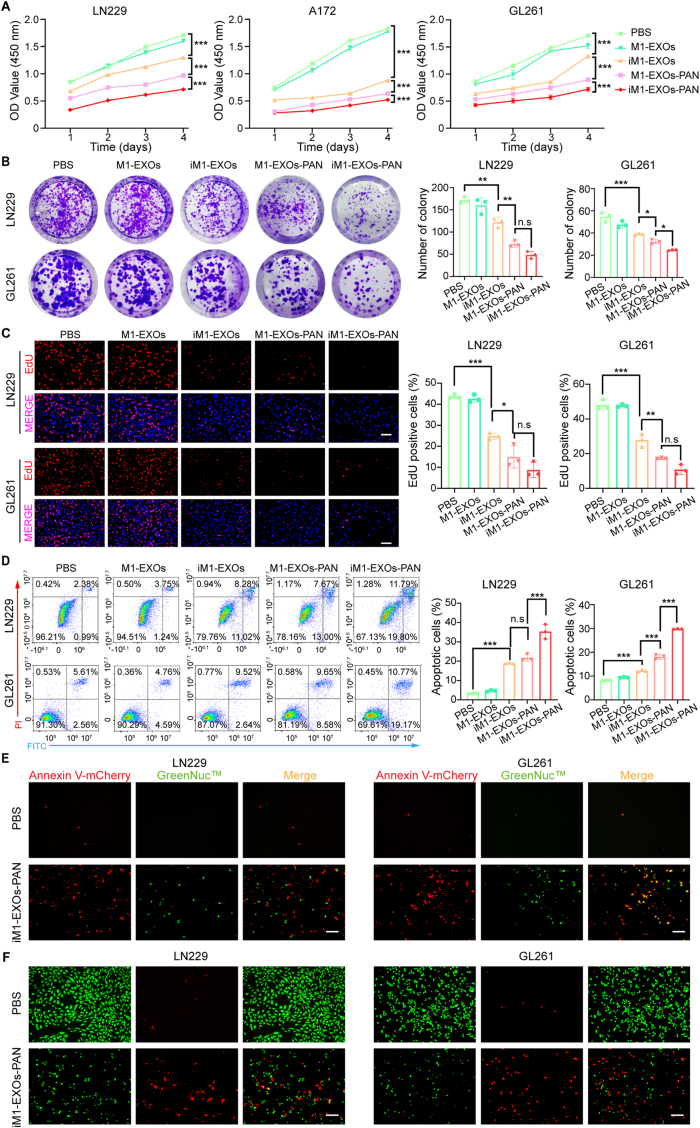
iM1-EXOs-PAN represses GB cells proliferation by inducing apoptosis. (A) Cell Counting Kit-8 assay was used to measure cell viability of LN229, A172, and GL261 at different time points (1, 2, 3, and 4 days). Data points are the OD450 values. (B) Representative images and statistical analysis for LN229 and GL261 in colony formation assay with indicated treatment. (C) Representative images and analysis of EdU-positive cells of LN229 and GL261 with indicated treatment. Scale bar, 50 μm. (D) Representative images and bar graphs representing quantitative analysis of LN229 and GL261 apoptosis assessed with flow cytometry. (E) Representative images of caspase-3 activity assay in LN229 and GL261 cells (red: Cells in apoptosis; green: caspase-3 positive cells). Scale bar, 50 μm. (F) Live-dead staining of LN229 and GL261 (green: live cells; red: dead cells). Scale bar, 50 μm. Panobinostat was administered at a concentration of 25 × 10^−9^ M. The data are presented as the means ± S.D. Two-way and one-way ANOVA were used for multigroup comparisons. n. s = not significant, ∗*P* < 0.05, ∗∗*P* < 0.01 and ∗∗∗*P* < 0.001. (For interpretation of the references to color in this figure legend, the reader is referred to the Web version of this article.)

Based on the STAT3-associated biological processes identified in Fig. S1 and panobinostat's known antitumor properties, we investigated the iM1-EXOs-PAN's *anti*-GB effects. CCK-8 assays revealed that M1 exosomes containing either si-STAT3 (iM1-EXOs) or panobinostat or both (iM1-EXOs-PAN) significantly inhibited the proliferation of GB cells compared to controls (Fig. 5A). Among the treatment groups, iM1-EXOs-PAN showed the most potent inhibition. These findings were corroborated by colony formation assays (Fig. 5B, S7A). Also, EdU assays performed on LN229, A172, and GL261 cells, showed that iM1-EXOs could halt the replication of GB cells. M1-EXOs-PAN exhibited a stronger inhibitory effect, and iM1-EXOs-PAN demonstrated the most potent suppression of cell replication (Fig. 5C, S7B). Although no statistically significant difference was observed between iM1-EXOs-PAN and M1-EXOs-PAN for LN229 and GL261 cells, the mean percentage of EdU-positive cells between the two groups differed by approximately 8 % and 6 %, respectivily (Fig. 5C). Furthermore, we performed flow cytometry analysis to evaluate the cytotoxic effects of M1-EXOs, iM1-EXOs, M1-EXOs-PAN, and iM1-EXOs-PAN on GB cells. The results indicated that both iM1-EXOs and M1-EXOs-PAN induced apoptosis in GB cells, with iM1-EXOs-PAN eliciting the most robust apoptotic response (Fig. 5D, S7C-D). The apoptotic effect was further verified by using the fluorescent caspase 3 marker GreenNuc™ (Fig. 5E, S7E). Live/Dead cell viability assays showed that while iM1-EXOs-PAN effectively induced death in GB cells, it demonstrated minimal toxicity toward NHA cells, indicating favorable therapeutic selectivity (Fig. 5F, S7F). Additionally, iM1-EXOs-PAN demonstrated superior inhibition of GB cell invasion and migration compared to other treatment groups (Fig. S8). To understand the molecular mechanisms underlying the antitumor effects of iM1-EXOs-PAN, GL261 cells treated with PBS or iM1-EXOs-PAN were collected and subjected to transcriptomic analysis. A total of 4246 differentially expressed genes were identified, including 3362 upregulated genes and 884 downregulated genes (|log2FC| > 1.0, P ≤ 0.05) (Fig. S9A–B). By KEGG enrichment analysis, the differentially expressed genes were found to play crucial roles in metabolic pathways, ferroptosis, cell adhesion molecules, and other pathways (Fig. S9C).

### 3.7 M2 to M1 macrophage repolarization by iM1-EXOs-PAN

**Fig. 6:**
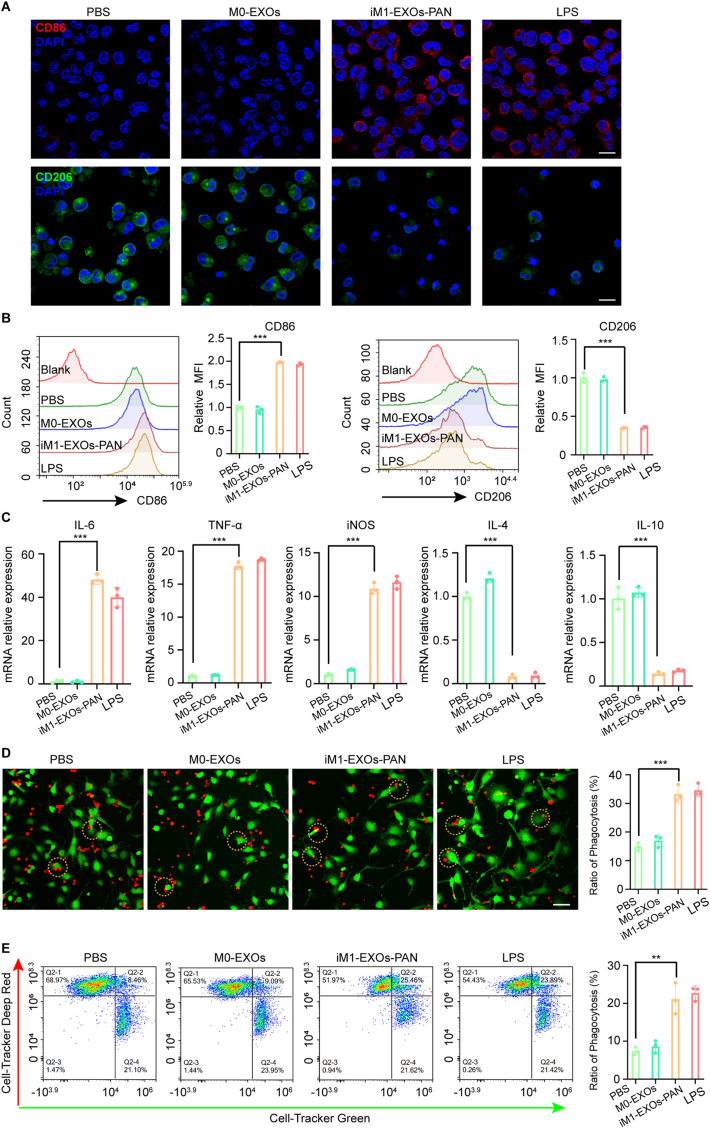
iM1-EXOs-PAN can effectively repolarize M2 macrophages to the M1 phenotype. (A) Confocal immunofluorescence microscopy images of CD86 (M1 macrophage marker) and CD206 (M2 macrophage marker) expression on M2 macrophages before and after treatments for 24 h. M0 EXOs (negative control), LPS (a commercial agent for M2 repolarization, positive control). Blue: DAPI-stained nucleus, red: CD86, green: CD206. Scale bar, 50 μm. (B) The expression of CD86 and CD206 on M2 macrophages was analyzed by flow cytometry before and after different treatments for 24 h. (C) The mRNA expression levels of inflammatory factors (IL-6, TNF-α, iNOS, IL-4, and IL-10) in M2 macrophages with different treatments. (D) GL261 phagocytosis assay of M2 macrophages with indicated treatments. Red: M2 macrophages, green: GL261 cells. Scale bar, 50 μm. (E) Flow cytometry analyzes the phagocytosis effect of M2 macrophages with the indicated treatments. The data are presented as the means ± S.D. Statistical significance was calculated using Student's *t*-test. ∗∗*P* < 0.01 and ∗∗∗*P* < 0.001. (For interpretation of the references to color in this figure legend, the reader is referred to the Web version of this article.)

Previous studies have shown the prevalence of M2 macrophages in the GB tumor microenvironment and their association with poor prognosis and immunosuppression [31,44]. It was shown that M1 exosomes have the potential to re-educate M2 into M1 macrophages, which can phagocytose tumor cells and effectively produce pro-inflammatory Th1-type cytokines [25,40,45]. Thus, we investigated the ability of iM1-EXOs-PAN to repolarize M2 macrophages. We compared the effects of PBS (blank control), primary macrophage-derived exosomes (M0-EXOs, negative control), iM1-EXOs-PAN, and LPS (positive control) on M2 macrophages over 24 h. Immunofluorescence analysis revealed that both LPS and iM1-EXOs-PAN treatments significantly increased expression of the M1 marker CD86 while decreasing expression of the M2 marker CD206 (Fig. 6A). Flow cytometry results further confirmed that iM1-EXOs-PAN can efficienttly revert macrophages from the M2 to the M1 phenotype (Fig. 6B). RT-qPCR analysis demonstrated upregulation of anti-tumor inflammatory cytokines (IL-6, TNF-α, iNOS) and concurrent downregulation of pro-tumor factors (IL-4, IL-10) (Fig. 6C), confirming successful M2 to M1 phenotype conversion with efficacy comparable to LPS treatment. To assess the functional consequences of repolarization, we evaluated phagocytic capacity using green-labeled GL261 tumor cells co-incubated with red-labeled M2 macrophages. While M2 macrophages treated with PBS or M0-EXOs showed minimal phagocytic activity, those treated with LPS or iM1-EXOs-PAN demonstrated robust tumor cell phagocytosis (Fig. 6D). Flow cytometry analysis provided quantitative validation of these observations (Fig. 6E). Furthermore, GL261 cells co-cultured with LPS- or iM1-EXOs-PAN-treated M2 macrophages exhibited significantly lower luciferase activity in tumor cells compared to the PBS or M0-EXOs groups (Fig. S10). These results demonstrated that iM1-EXOs-PAN can regulate repolarization of M2 macrophages, which in turn enhanced macrophage phagocytosis of GB cells.

### 3.8 In vivo brain tumor targeting and anti-tumor efficacy

**Fig. 7:**
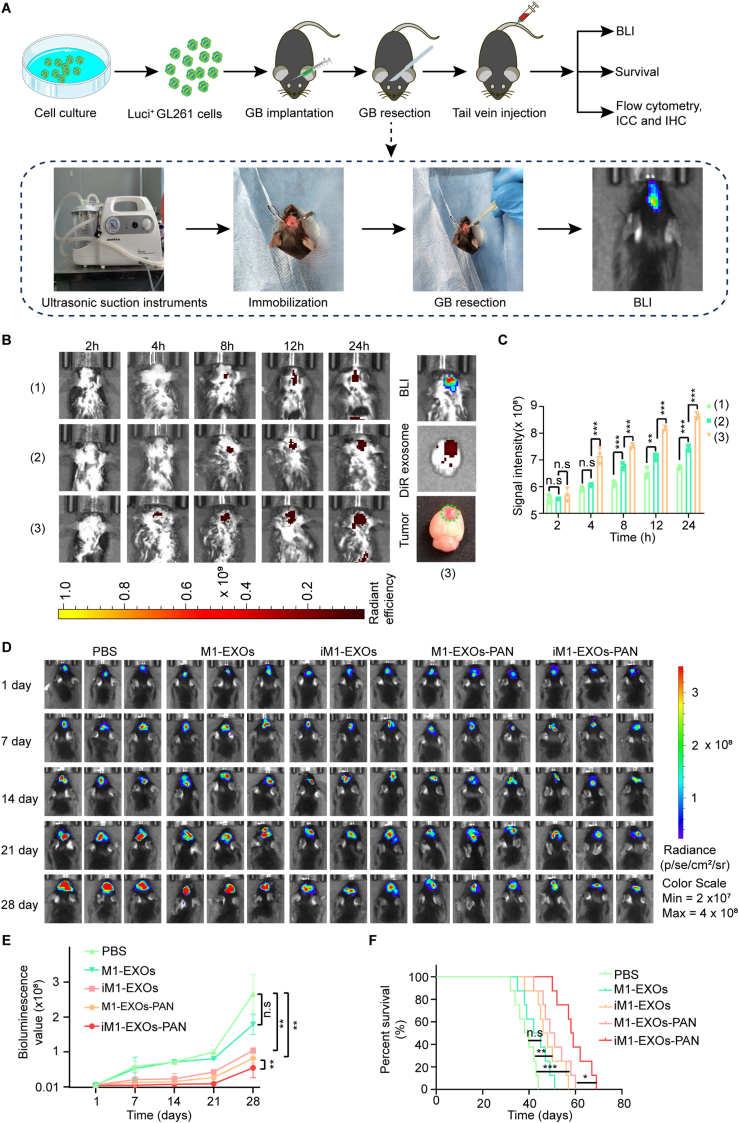
iM1-EXOs-PAN are recruited to the brain tumor and mediate an anti-tumor effect. (A) Schematic illustration of iM1-EXOs-PAN-mediated anticancer drug delivery for inhibiting GB recurrence after surgical tumor removal. (B–C) Real-time in vivo fluorescence imaging and quantification of the normal mice (1), GL261-bearing mice (2), and surgically treated GL261-bearing mice (3) after intravenous administration of iM1-EXOs-PAN. (D–E) Representative in vivo bioluminescent images and quantification of fluorescence signals in postoperative treatment of GL261^luc +^ GB-bearing C57BL/6 mice following PBS, M1-EXOs, iM1-EXOs, M1-EXOs-PAN, and iM1-EXOs-PAN treatment. 1 day: the first day after resection of the GB. (F) Kaplan-Meier survival analysis of postoperative GB-bearing C57BL/6 mice. One-way ANOVA was used for multigroup comparisons. The survival data were analyzed by using the log-rank (Mantel–Cox) test. n. s = not significant, ∗*P* < 0.05, ∗∗*P* < 0.01 and ∗∗∗*P* < 0.001. Time: survival time of mice after GB resection.

We used an *in situ* mouse glioma model by intracranially injecting GL261^luci^ cells, followed by surgical resection at day 8 post-implantation to simulate clinical conditions (Fig. 7A) as described previously [29]. To evaluate biodistribution, we monitored DiR-labeled iM1-EXOs-PAN (iM1-EXOs-PAN/DiR) following intravenous administration in three groups: normal mice, GL261-bearing mice, and surgically treated GL261-bearing mice. The surgically treated group showed significantly greater brain-targeting capability than both control groups, with fluorescence intensity increasing progressively over 24 h (Fig. 7B and C). This improved targeting effect was attributed to surgery-amplified inflammatory responses, enabling more effective targeting of infiltrating glioma cells. Therapeutic efficacy was assessed through bioluminescence monitoring in GL261^luci^-bearing mice following various treatments. While control PBS-treated tumors showed extensive growth, M1-EXOs treatment demonstrated modest inhibition of growth. Both iM1-EXOs and M1-EXOs-PAN showed enhanced tumor suppression, with iM1-EXOs-PAN exhibiting the most potent inhibitory effect and lowest tumor bioluminescence (Fig. 7D and E). Kaplan-Meier survival analysis revealed median survival times of 39, 43.5, 47, 50, and >58 days for PBS, M1-EXOs, iM1-EXOs, M1-EXOs-PAN, and iM1-EXOs-PAN treatment groups, respectively (Fig. 7F). IHC analysis showed that iM1-EXOs-PAN treatment most effectively reduced Ki67 and STAT3 expression while increasing Bax expression in GB tissues (Fig. S11A–B). TUNEL assay revealed the highest proportion of apoptotic cells in the iM1-EXOs-PAN group (Fig. S12). These data strongly confirm that the co-delivery strategy significantly inhibited GB recurrence.

### 3.9 Tumor microenvironment remodeling by iM1-EXOs-PAN

**Fig. 8:**
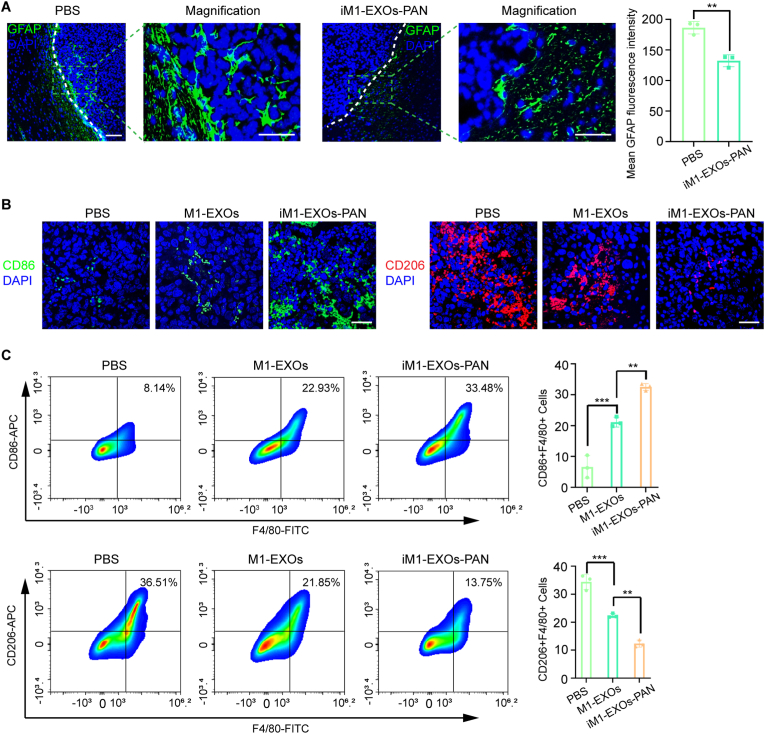
iM1-EXOs-PAN can remodel the tumor microenvironment. (A) Representative GFAP (Green) fluorescent images of iM1-EXOs-PAN inhibition of NHA activation around tumors. A bar plot represents the mean fluorescence intensity of GFAP expression in astrocytes. Scale bar, 50 μm. (B) Representative fluorescent images of PBS, M1-EXOs, and iM1-EXOs-PAN in reducing M2 macrophage infiltration and increasing M1 macrophage infiltration in recurrent GB tissues. Scale bar, 50 μm. (C) Flow cytometry analysis of F4/80-CD86 and F4/80-CD206 cells in recurrent GB tissues after PBS, M1-EXOs, and iM1-EXOs-PAN treatment. The data are presented as the means ± S.D. Statistical significance in (A) was calculated using Student's *t*-test. One-way ANOVA was used for multigroup comparisons. ∗∗*P* < 0.01 and ∗∗∗*P* < 0.001. (For interpretation of the references to color in this figure legend, the reader is referred to the Web version of this article.)

We further explored the role of iM1-EXOs-PAN on the tumor microenvironment. Immunofluorescence analysis of tissue sections revealed significantly reduced numbers of GFAP-expressing activated astrocytes surrounding recurrent tumors following iM1-EXOs-PAN treatment (Fig. 8A). Additionally, the treatment significantly altered the tumor-associated macrophage profile, reducing M2 macrophage infiltration while increasing M1 macrophage presence in recurrent GB tissues (Fig. 8B and C, S13). These findings align with previous reports showing that STAT3 silencing reduces M2-polarized macrophages and greatly increases M1-polarized macrophages [46]. Collectively, our results demonstrate that our dual targeted therapy produces combined effects through the modification of cellular crosstalk within the tumor microenvironment, thereby enhancing overall antitumor efficacy.

## 4 Discussion

GBs show resistance to conventional therapies, even after precise surgical intervention, where their infiltrative growth pattern and complex microenvironmental interactions represent a significant problem. Effective treatments for recurrent GB necessitate a multidimensional therapeutic strategy that directly targets tumor cells and modifies the surrounding TME [47,48]. STAT3 is a key signaling pathway involved in the progression and modulation of the tumor microenvironment across multiple cancer types. In GB, STAT3 contributes to malignant behavior, in part, by regulating mechanisms of immune evasion [[49], [50], [51]]. Accumulating evidence indicates that the JAK/STAT3 pathway plays a vital role in the pathogenesis of several malignancies. The primary mode of STAT3 activation involves cellular stimulation by cytokines (such as interferons and interleukins) or growth factors (like epidermal growth factor (EGF)). This activation is mediated by phosphorylation at tyrosine 705 (Y705) and serine 727 (S727) [52,53]. Furthermore, the Notch pathway has been reported to be activated downstream of STAT3 activation. Delta-like 4 (DLL4) and Jagge1 (Jag1) Notch ligands were activated by STAT3 phosphorylated at serine 727 to promote human embryonic stem cell survival [54]. In this study, we demonstrated that STAT3 knockdown can inhibit the proliferation, invasion, and migration of GB, block the promotion of activated NHA on GB, and inhibit intercellular crosstalk. These results suggest that targeting STAT3 could be an innovative treatment option against both GB cells and active astrocytes in the TME. Our study further highlights the crucial role of STAT3 signaling in GB progression and immune evasion. Additionally, the analysis of postoperative relapse cases revealed sustained STAT3 activation in residual tumor cells following surgical intervention. We also established STAT3's multifaceted influence on GB cellular behavior, including the regulation of proliferation, migration, and cell death pathways. Notably, we uncovered a previously underappreciated mechanism in which GB cells with constitutively active STAT3 initiate and sustain astrocyte activation via STAT3-dependent signaling. This finding is particularly significant in the context of surgical intervention, as our co-culture experiments demonstrated that activated astrocytes enhance both the proliferation and invasion of GB cells.

The iM1-EXOs-PAN delivery system developed in this study addresses several critical challenges in GB therapy. First, our in vitro BBB model demonstrates that iM1-EXOs-PAN efficiently crosses the endothelial barrier, particularly in response to inflammatory stimuli. This enhanced penetration under fMLP stimulation suggests that the system can exploit inflammation-mediated trafficking pathways, a property especially relevant in post-surgical scenarios where inflammatory responses are elevated. As many studies indicate that macrophages exhibit substantial plasticity and can be polarized to M1 anti-tumoral or M2 pro-tumoral phenotype in response to microenvironmental changes [55,56], it is conceivable that the use of macrophage-derived exosomes instead of macrophages themselves could eliminate the potential risk of an M1 to M2 switch during tumor progression, while preserving distinct macrophage functions, such as inflammatory chemotaxis and BBB penetration. Second, the system also successfully overcomes the poor water solubility and delivery challenges associated with panobinostat. Notably, this therapeutic cargo does not compromise the integrity of the delivery vehicle, as evidenced by the preservation of exosome morphology and a consistent size distribution of approximately 110 nm after drug loading. The co-delivery of panobinostat and siSTAT3 demonstrates remarkable combined effects. Flow cytometry and TUNEL assays reveal enhanced apoptosis induction compared to single-agent treatments. RNA sequencing further reveals that differentially expressed genes are enriched in metabolic pathways, ferroptosis, cell adhesion molecules, and other pathways. In addition, the system effectively modulates the tumor microenvironment, significantly reducing GFAP-expressing reactive astrocytes while favorably redistributing M1/M2 macrophage populations. These combined effects result in increased survival outcomes, with a median survival time extended beyond 58 days compared to 39 days in the control groups.

Another innovative aspect of this therapeutic approach is the use of engineered M1 macrophage-derived exosomes as delivery vehicles. This choice was triggered by a prior transcriptomic study showing a declined tumor purity over time and increased tumor-associated macrophage infiltration [57]. Also, the predominance of M2-like macrophages in GB samples, primarily derived from peripheral blood, provided a strategic opportunity for intervention [58,59]. Therefore, our engineered M1 exosomes demonstrated dual functionality: serving as efficient delivery vehicles while simultaneously promoting the conversion of tumor-promoting M2 macrophages to tumor-suppressing M1 phenotypes. In this context, the temporal progression of therapeutic effects, from initial tumor targeting to sustained microenvironment modification, represents a significant advance over conventional delivery approaches. This comprehensive impact on both tumor cells and their microenvironment suggests the potential for improved treatment outcomes in GB. It should be acknowledged that the problem of the formation of protein coronas may represent a problem when it comes to therapeutic delivery of exosomes. However, as shown in Fig. 7B, a clear accumulation of iM1-EXOs-PAN was seen in the brain. Further studies are, however, needed to assess if this accumulation can be optimized.

Although exosomes have demonstrated significant efficacy in targeted tumor therapy, their interactions with recipient cells depend on specific proteins, lipids, and glycans on their surfaces, as well as their overall negative charge. The uptake of exosomes can be selective or non-specific [60]. It should be emphasized that understanding how exosomes naturally achieve lysosomal degradation escape is crucial for designing effective drug delivery systems. Over the years, different mechanisms have been proposed and investigated that enable exosomes-based drug delivery systems to escape the lysosomal entrapment, such as membrane fusion, pore formation, “Back-fusion” and pH buffering [61,62]. The significance of these lysosomal escape mechanisms may be influenced by factors such as the cellular microenvironment, exosome subtypes, the cargos, and target cell types. Exosomes may also naturally incorporate some lysosomal escape mechanisms, which remain unknown to date. Further research is essential for fully understanding these complex processes and their implications for the design of next-generation exosomes-based drug delivery systems [62].

Although our system demonstrates promising therapeutic efficacy, the long-term stability of reprogrammed macrophage phenotypes remains to be investigated. The dynamic nature of the tumor microenvironment suggests that sustained M2 to M1 conversion may face challenges over extended periods. Additionally, collected evidence indicates that STAT3 is typically hyperactivated in TME cells, including T cells, NK cells, Treg cells, dendritic cells, and cancer-associated fibroblasts. The hyperactivation of STAT3 in these TME cells may exert a significant impact on the anti-tumor immune response through multiple mechanisms [[63], [64], [65]]. In this context, therapies targeting STAT3 have shown great potential in reversing the immunosuppressive microenvironment [46,66]. Hence, we are preparing to investigate further the effects of iM1-EXOs-PAN on immune cells, including CD4^+^ T cells, CD8^+^ T cells, and Treg cells, etc. In vivo. The current study focused primarily on immediate and short-term responses. However, the longer-term studies will be essential for understanding the durability of therapeutic effects. The ability of the current system to modulate the tumor microenvironment suggests potential synergies with immunotherapies or other targeted treatments, which might complement or enhance current treatment modalities that could potentially lead to more effective therapeutic strategies.

In conclusion, this “one-two punch" therapeutic strategy represents a promising approach for addressing GB recurrence by simultaneously targeting multiple aspects of the disease. The demonstrated ability to cross the BBB, target tumor cells and astrocytes, and modulate the immune environment provides a strong foundation for future clinical development. While significant challenges remain to be addressed, the robust therapeutic effects observed in our study suggest considerable potential for improving treatment outcomes in GB patients.

## CRediT authorship contribution statement

**Xuemeng Liu:** Writing – original draft, Conceptualization. **Yaotian Hu:** Writing – original draft, Funding acquisition, Data curation. **Yan Zhang:** Methodology. **Chang Liu:** Methodology. **Jingwen Wu:** Methodology. **Ruiqi Zhao:** Methodology. **Zhiyi Xue:** Methodology. **Wenjing Zhou:** Methodology. **Xiaofei Liu:** Methodology. **Hrvoje Miletic:** Methodology. **Yongli Gao:** Methodology. **Chen Qiu:** Supervision, Methodology. **Jian Wang:** Writing – review & editing, Supervision, Funding acquisition, Conceptualization.

## Ethics approval and consent to participate

All animal experiments were approved by Qilu Hospital of Shandong University. Human glioma tissue samples were obtained from surgeries performed on patients at Qilu Hospital. All patients enrolled provided written informed consent.

## Funding

This work was supported by the China National “111” Project (B20058), the Special Foundation for Taishan Scholars (tshw201502056), the 10.13039/501100007129Shandong Provincial Natural Science Foundation (ZR2023QH524 and ZR2024MH040), the 10.13039/501100012217Xuzhou Medical University Excellent Talent Foundation (XYFY202429), Linyi Medical and 10.13039/501100000724Health Foundation (202501) and 10.13039/501100002858China Postdoctoral Science Foundation (2020M672072).

## Declaration of competing interest

The authors declare that they have no known competing financial interests or personal relationships that could have appeared to influence the work reported in this paper.

## Data Availability

All data for the analysis of our study are available from the corresponding authors upon reasonable request.

## References

[1] Cuddapah V.A., Robel S., Watkins S., Sontheimer H. (2014). A neurocentric perspective on glioma invasion. Nat. Rev. Neurosci..

[2] Li C., Wang S., Yan J.-L., Torheim T., Boonzaier N.R., Sinha R., Matys T., Markowetz F., Price S.J. (2019). Characterizing tumor invasiveness of glioblastoma using multiparametric magnetic resonance imaging. J. Neurosurg..

[3] Ratel D., van der Sanden B., Wion D. (2016). Glioma resection and tumor recurrence: back to semmelweis. Neuro Oncol..

[4] Knudsen A.M., Halle B., Cédile O., Burton M., Baun C., Thisgaard H., Anand A., Hubert C., Thomassen M., Michaelsen S.R., Olsen B.B., Dahlrot R.H., Bjerkvig R., Lathia J.D., Kristensen B.W. (2022). Surgical resection of glioblastomas induces pleiotrophin-mediated self-renewal of glioblastoma stem cells in recurrent tumors. Neuro Oncol..

[5] Claes A., Idema A.J., Wesseling P. (2007). Diffuse glioma growth: a guerilla war. Acta Neuropathol..

[6] Huang-Hobbs E., Cheng Y.-T., Ko Y., Luna-Figueroa E., Lozzi B., Taylor K.R., McDonald M., He P., Chen H.-C., Yang Y., Maleki E., Lee Z.-F., Murali S., Williamson M.R., Choi D., Curry R., Bayley J., Woo J., Jalali A., Monje M., Noebels J.L., Harmanci A.S., Rao G., Deneen B. (2023). Remote neuronal activity drives glioma progression through SEMA4F. Nature.

[7] Bazargani N., Attwell D. (2016). Astrocyte calcium signaling: the third wave. Nat. Neurosci..

[8] Khakh B.S., Sofroniew M.V. (2015). Diversity of astrocyte functions and phenotypes in neural circuits. Nat. Neurosci..

[9] Pekny M., Pekna M. (2014). Astrocyte reactivity and reactive astrogliosis: costs and benefits. Physiol. Rev..

[10] Vandenbark A.A., Offner H., Matejuk S., Matejuk A. (2021). Microglia and astrocyte involvement in neurodegeneration and brain cancer. J. Neuroinflammation.

[11] Placone A.L., Quiñones-Hinojosa A., Searson P.C. (2016). The role of astrocytes in the progression of brain cancer: complicating the picture of the tumor microenvironment. Tumour Biol.

[12] Okolie O., Bago J.R., Schmid R.S., Irvin D.M., Bash R.E., Miller C.R., Hingtgen S.D. (2016). Reactive astrocytes potentiate tumor aggressiveness in a murine glioma resection and recurrence model. Neuro Oncol..

[13] Henrik Heiland D., Ravi V.M., Behringer S.P., Frenking J.H., Wurm J., Joseph K., Garrelfs N.W.C., Strähle J., Heynckes S., Grauvogel J., Franco P., Mader I., Schneider M., Potthoff A.-L., Delev D., Hofmann U.G., Fung C., Beck J., Sankowski R., Prinz M., Schnell O. (2019). Tumor-associated reactive astrocytes aid the evolution of immunosuppressive environment in glioblastoma. Nat. Commun..

[14] Zhang C.-B., Wang Z.-L., Liu H.-J., Wang Z., Jia W. (2023). Characterization of tumor-associated reactive astrocytes in gliomas by single-cell and bulk tumor sequencing. Front. Neurol..

[15] Wang K.-N., Zhou K., Zhong N.-N., Cao L.-M., Li Z.-Z., Xiao Y., Wang G.-R., Huo F.-Y., Zhou J.-J., Liu B., Bu L.-L. (2024). Enhancing cancer therapy: the role of drug delivery systems in STAT3 inhibitor efficacy and safety. Life Sci..

[16] Gupta M., Han J.J., Stenson M., Wellik L., Witzig T.E. (2012). Regulation of STAT3 by histone deacetylase-3 in diffuse large B-cell lymphoma: implications for therapy. Leukemia.

[17] Lee E.Q., Reardon D.A., Schiff D., Drappatz J., Muzikansky A., Grimm S.A., Norden A.D., Nayak L., Beroukhim R., Rinne M.L., Chi A.S., Batchelor T.T., Hempfling K., McCluskey C., Smith K.H., Gaffey S.C., Wrigley B., Ligon K.L., Raizer J.J., Wen P.Y. (2015). Phase II study of panobinostat in combination with bevacizumab for recurrent glioblastoma and anaplastic glioma. Neuro Oncol..

[18] García-Fernández J., de la Fuente Freire M. (2023). Exosome-like systems: nanotechnology to overcome challenges for targeted cancer therapies. Cancer Lett..

[19] Rufino-Ramos D., Albuquerque P.R., Carmona V., Perfeito R., Nobre R.J., Pereira de Almeida L. (2017). Extracellular vesicles: novel promising delivery systems for therapy of brain diseases. J. Contr. Release.

[20] Wang J., Tang W., Yang M., Yin Y., Li H., Hu F., Tang L., Ma X., Zhang Y., Wang Y. (2021). Inflammatory tumor microenvironment responsive neutrophil exosomes-based drug delivery system for targeted glioma therapy. Biomaterials.

[21] Zhang W., Wang M., Tang W., Wen R., Zhou S., Lee C., Wang H., Jiang W., Delahunty I.M., Zhen Z., Chen H., Chapman M., Wu Z., Howerth E.W., Cai H., Li Z., Xie J. (2018). Nanoparticle-laden macrophages for tumor-tropic drug delivery. Adv Mater.

[22] Ying K., Zhu Y., Wan J., Zhan C., Wang Y., Xie B., Xu P., Pan H., Wang H. (2023). Macrophage membrane-biomimetic adhesive polycaprolactone nanocamptothecin for improving cancer-targeting efficiency and impairing metastasis. Bioact. Mater..

[23] Xie J., Wang J., Niu G., Huang J., Chen K., Li X., Chen X. (2010). Human serum albumin coated iron oxide nanoparticles for efficient cell labeling. Chem. Commun..

[24] Nie W., Wu G., Zhang J., Huang L.-L., Ding J., Jiang A., Zhang Y., Liu Y., Li J., Pu K., Xie H.-Y. (2020). Responsive exosome nano-bioconjugates for synergistic cancer therapy. Angew Chem. Int. Ed. Engl..

[25] Wang P., Wang H., Huang Q., Peng C., Yao L., Chen H., Qiu Z., Wu Y., Wang L., Chen W. (2019). Exosomes from M1-Polarized macrophages enhance paclitaxel antitumor activity by activating macrophages-mediated inflammation. Theranostics.

[26] Choo Y.W., Kang M., Kim H.Y., Han J., Kang S., Lee J.-R., Jeong G.-J., Kwon S.P., Song S.Y., Go S., Jung M., Hong J., Kim B.-S. (2018). M1 macrophage-derived nanovesicles potentiate the anticancer efficacy of immune checkpoint inhibitors. ACS Nano.

[27] Baek S., Jeon M., Jung H.N., Lee W., Hwang J.-E., Lee J.S., Choi Y., Im H.-J. (2022). M1 macrophage-derived exosome-mimetic nanovesicles with an enhanced cancer targeting ability. ACS Appl. Bio Mater..

[28] Qian B.-Z., Li J., Zhang H., Kitamura T., Zhang J., Campion L.R., Kaiser E.A., Snyder L.A., Pollard J.W. (2011). CCL2 recruits inflammatory monocytes to facilitate breast-tumour metastasis. Nature.

[29] Zhu H., Leiss L., Yang N., Rygh C.B., Mitra S.S., Cheshier S.H., Weissman I.L., Huang B., Miletic H., Bjerkvig R., Enger P.Ø., Li X., Wang J. (2017). Surgical debulking promotes recruitment of macrophages and triggers glioblastoma phagocytosis in combination with CD47 blocking immunotherapy. Oncotarget.

[30] Xiang X., Wang J., Lu D., Xu X. (2021). Targeting tumor-associated macrophages to synergize tumor immunotherapy. Signal Transduct. Targeted Ther..

[31] Zhang H., Liu L., Liu J., Dang P., Hu S., Yuan W., Sun Z., Liu Y., Wang C. (2023). Roles of tumor-associated macrophages in anti-PD-1/PD-L1 immunotherapy for solid cancers. Mol. Cancer.

[32] Ma X., Yao M., Gao Y., Yue Y., Li Y., Zhang T., Nie G., Zhao X., Liang X. (2022). Functional immune cell‐derived exosomes engineered for the trilogy of radiotherapy sensitization. Adv. Sci. (Weinh.).

[33] Ding J., Zhang Y., Cai X., Zhang Y., Yan S., Wang J., Zhang S., Yin T., Yang C., Yang J. (2021). Extracellular vesicles derived from M1 macrophages deliver miR-146a-5p and miR-146b-5p to suppress trophoblast migration and invasion by targeting TRAF6 in recurrent spontaneous abortion. Theranostics.

[34] Zhou W., Zhou Y., Chen X., Ning T., Chen H., Guo Q., Zhang Y., Liu P., Zhang Y., Li C., Chu Y., Sun T., Jiang C. (2021). Pancreatic cancer-targeting exosomes for enhancing immunotherapy and reprogramming tumor microenvironment. Biomaterials.

[35] Haney M.J., Klyachko N.L., Zhao Y., Gupta R., Plotnikova E.G., He Z., Patel T., Piroyan A., Sokolsky M., Kabanov A.V., Batrakova E.V. (2015). Exosomes as drug delivery vehicles for Parkinson's disease therapy. J. Contr. Release.

[36] Li B., Chen X., Qiu W., Zhao R., Duan J., Zhang S., Pan Z., Zhao S., Guo Q., Qi Y., Wang W., Deng L., Ni S., Sang Y., Xue H., Liu H., Li G. (2022). Synchronous disintegration of ferroptosis defense axis via engineered exosome-conjugated magnetic nanoparticles for glioblastoma therapy. Adv. Sci. (Weinh.).

[37] Xue J., Zhao Z., Zhang L., Xue L., Shen S., Wen Y., Wei Z., Wang L., Kong L., Sun H., Ping Q., Mo R., Zhang C. (2017). Neutrophil-mediated anticancer drug delivery for suppression of postoperative malignant glioma recurrence. Nat. Nanotechnol..

[38] Liu W., Chen Y., Meng J., Wu M., Bi F., Chang C., Li H., Zhang L. (2018). Ablation of caspase-1 protects against TBI-Induced pyroptosis in vitro and in vivo. J. Neuroinflammation.

[39] Wu Q., Gao C., Wang H., Zhang X., Li Q., Gu Z., Shi X., Cui Y., Wang T., Chen X., Wang X., Luo C., Tao L. (2018). Mdivi-1 alleviates blood-brain barrier disruption and cell death in experimental traumatic brain injury by mitigating autophagy dysfunction and mitophagy activation. Int. J. Biochem. Cell Biol..

[40] Ding J., Lu G., Nie W., Huang L.-L., Zhang Y., Fan W., Wu G., Liu H., Xie H.-Y. (2021). Self-activatable photo-extracellular vesicle for synergistic trimodal anticancer therapy. Adv Mater.

[41] Brandao M., Simon T., Critchley G., Giamas G. (2019). Astrocytes, the rising stars of the glioblastoma microenvironment. Glia.

[42] Tyzack G.E., Sitnikov S., Barson D., Adams-Carr K.L., Lau N.K., Kwok J.C., Zhao C., Franklin R.J.M., Karadottir R.T., Fawcett J.W., Lakatos A. (2014). Astrocyte response to motor neuron injury promotes structural synaptic plasticity via STAT3-regulated TSP-1 expression. Nat. Commun..

[43] O'Callaghan J.P., Kelly K.A., VanGilder R.L., Sofroniew M.V., Miller D.B. (2014). Early activation of STAT3 regulates reactive astrogliosis induced by diverse forms of neurotoxicity. PLoS One.

[44] Klemm F., Maas R.R., Bowman R.L., Kornete M., Soukup K., Nassiri S., Brouland J.-P., Iacobuzio-Donahue C.A., Brennan C., Tabar V., Gutin P.H., Daniel R.T., Hegi M.E., Joyce J.A. (2020). Interrogation of the microenvironmental landscape in brain tumors reveals disease-specific alterations of immune cells. Cell.

[45] Gunassekaran G.R., Poongkavithai Vadevoo S.M., Baek M.-C., Lee B. (2021). M1 macrophage exosomes engineered to foster M1 polarization and target the IL-4 receptor inhibit tumor growth by reprogramming tumor-associated macrophages into M1-like macrophages. Biomaterials.

[46] Peng Z., Zhao T., Gao P., Zhang G., Wu X., Tian H., Qu M., Tan X., Zhang Y., Zhao X., Qi X. (2024). Tumor-derived extracellular vesicles enable tumor tropism chemo-genetherapy for local immune activation in triple-negative breast cancer. ACS Nano.

[47] Darmanis S., Sloan S.A., Croote D., Mignardi M., Chernikova S., Samghababi P., Zhang Y., Neff N., Kowarsky M., Caneda C., Li G., Chang S.D., Connolly I.D., Li Y., Barres B.A., Gephart M.H., Quake S.R. (2017). Single-cell RNA-seq analysis of infiltrating neoplastic cells at the migrating front of human glioblastoma. Cell Rep..

[48] Varn F.S., Johnson K.C., Martinek J., Huse J.T., Nasrallah M.P., Wesseling P., Cooper L.A.D., Malta T.M., Wade T.E., Sabedot T.S., Brat D., Gould P.V., Wöehrer A., Aldape K., Ismail A., Sivajothi S.K., Barthel F.P., Kim H., Kocakavuk E., Ahmed N., White K., Datta I., Moon H.-E., Pollock S., Goldfarb C., Lee G.-H., Garofano L., Anderson K.J., Nehar-Belaid D., Barnholtz-Sloan J.S., Bakas S., Byrne A.T., D'Angelo F., Gan H.K., Khasraw M., Migliozzi S., Ormond D.R., Paek S.H., Van Meir E.G., Walenkamp A.M.E., Watts C., Weiss T., Weller M., Palucka K., Stead L.F., Poisson L.M., Noushmehr H., Iavarone A., Verhaak R.G.W. (2022). GLASS consortium, glioma progression is shaped by genetic evolution and microenvironment interactions. Cell.

[49] Fu W., Hou X., Dong L., Hou W. (2023). Roles of STAT3 in the pathogenesis and treatment of glioblastoma. Front. Cell Dev. Biol..

[50] Zou S., Tong Q., Liu B., Huang W., Tian Y., Fu X. (2020). Targeting STAT3 in cancer immunotherapy. Mol. Cancer.

[51] Hu G., Fang Y., Xu H., Wang G., Yang R., Gao F., Wei Q., Gu Y., Zhang C., Qiu J., Gao N., Wen Q., Qiao H. (2023). Identification of cytochrome P450 2E1 as a novel target in glioma and development of its inhibitor as an anti-tumor agent. Adv. Sci. (Weinh.).

[52] Johnson D.E., O'Keefe R.A., Grandis J.R. (2018). Targeting the IL-6/JAK/STAT3 signalling axis in cancer. Nat. Rev. Clin. Oncol..

[53] Lv G., Li X., Deng H., Zhang J., Gao X. (2024). Regulatory mechanisms of STAT3 in GBM and its impact on TMZ resistance. Curr. Mol. Pharmacol..

[54] Zhang G., Tanaka S., Jiapaer S., Sabit H., Tamai S., Kinoshita M., Nakada M. (2020). RBPJ contributes to the malignancy of glioblastoma and induction of proneural-mesenchymal transition via IL-6-STAT3 pathway. Cancer Sci..

[55] Sheu K.M., Hoffmann A. (2022). Functional hallmarks of healthy macrophage responses: their regulatory basis and disease relevance. Annu. Rev. Immunol..

[56] Liu S., Zhang H., Li Y., Zhang Y., Bian Y., Zeng Y., Yao X., Wan J., Chen X., Li J., Wang Z., Qin Z. (2021). S100A4 enhances protumor macrophage polarization by control of PPAR-γ-dependent induction of fatty acid oxidation. J. Immunother. Cancer.

[57] Hoogstrate Y., Draaisma K., Ghisai S.A., van Hijfte L., Barin N., de Heer I., Coppieters W., van den Bosch T.P.P., Bolleboom A., Gao Z., Vincent A.J.P.E., Karim L., Deckers M., Taphoorn M.J.B., Kerkhof M., Weyerbrock A., Sanson M., Hoeben A., Lukacova S., Lombardi G., Leenstra S., Hanse M., Fleischeuer R.E.M., Watts C., Angelopoulos N., Gorlia T., Golfinopoulos V., Bours V., van den Bent M.J., Robe P.A., French P.J. (2023). Transcriptome analysis reveals tumor microenvironment changes in glioblastoma. Cancer Cell.

[58] Wang S., Wang J., Chen Z., Luo J., Guo W., Sun L., Lin L. (2024). Targeting M2-like tumor-associated macrophages is a potential therapeutic approach to overcome antitumor drug resistance. npj Precis. Oncol..

[59] Wang X., Ding H., Li Z., Peng Y., Tan H., Wang C., Huang G., Li W., Ma G., Wei W. (2022). Exploration and functionalization of M1-macrophage extracellular vesicles for effective accumulation in glioblastoma and strong synergistic therapeutic effects. Signal Transduct. Targeted Ther..

[60] György B., Hung M.E., Breakefield X.O., Leonard J.N. (2015). Therapeutic applications of extracellular vesicles: clinical promise and open questions. Annu. Rev. Pharmacol. Toxicol..

[61] Hagedorn L., Jürgens D.C., Merkel O.M., Winkeljann B. (2024). Endosomal escape mechanisms of extracellular vesicle-based drug carriers: lessons for lipid nanoparticle design. Extracell Vesicles Circ Nucl Acids.

[62] O'Brien K., Breyne K., Ughetto S., Laurent L.C., Breakefield X.O. (2020). RNA delivery by extracellular vesicles in Mammalian cells and its applications. Nat. Rev. Mol. Cell Biol..

[63] Wang H., Liang Y., Liu Z., Zhang R., Chao J., Wang M., Liu M., Qiao L., Xuan Z., Zhao H., Lu L. (2024). POSTN+ cancer-associated fibroblasts determine the efficacy of immunotherapy in hepatocellular carcinoma. J. Immunother. Cancer.

[64] Ma S., Sun B., Duan S., Han J., Barr T., Zhang J., Bissonnette M.B., Kortylewski M., He C., Chen J., Caligiuri M.A., Yu J. (2023). YTHDF2 orchestrates tumor-associated macrophage reprogramming and controls antitumor immunity through CD8+ T cells. Nat. Immunol..

[65] Zhou J., Tison K., Zhou H., Bai L., Acharyya R.K., McEachern D., Metwally H., Wang Y., Pitter M., Choi J.E., Vatan L., Liao P., Yu J., Lin H., Jiang L., Wei S., Gao X., Grove S., Parolia A., Cieslik M., Kryczek I., Green M.D., Lin J.-X., Chinnaiyan A.M., Leonard W.J., Wang S., Zou W. (2025). STAT5 and STAT3 balance shapes dendritic cell function and tumour immunity. Nature.

[66] Hu Y., Dong Z., Liu K. (2024). Unraveling the complexity of STAT3 in cancer: molecular understanding and drug discovery. J. Exp. Clin. Cancer Res..

