# Supplementary material for: Co-delivery of panobinostat and siSTAT3 using engineered M1 exosomes to establish a one-two punch therapeutic strategy for glioblastoma recurrence
Source: Mater Today Bio. 2025 Dec 13;36:102680. doi: 10.1016/j.mtbio.2025.102680 (PMC12813245; doi:10.1016/j.mtbio.2025.102680)
Supplement: Multimedia component 1 [file mmc1.docx]

**Supplementary Materials**


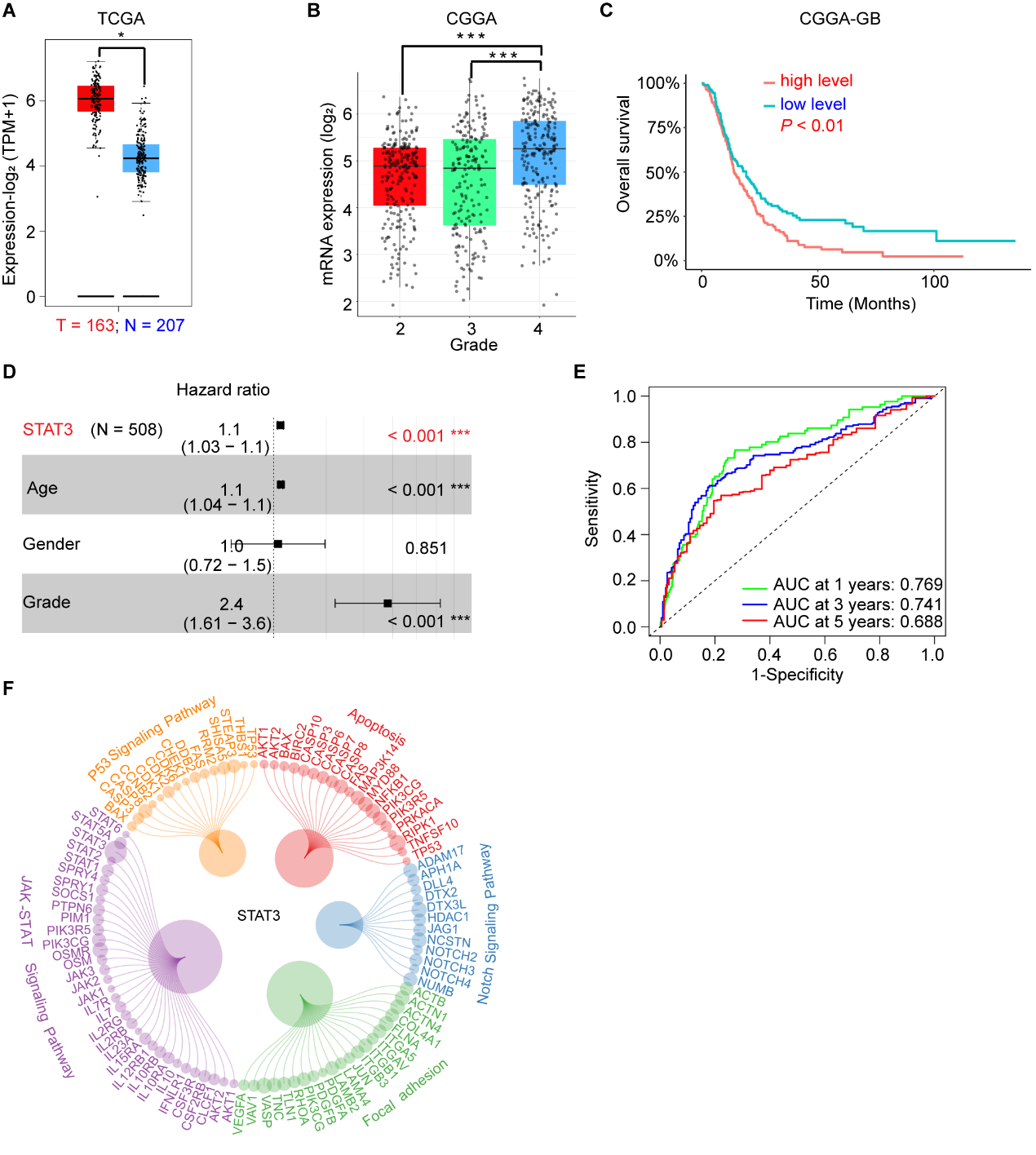


**Fig. S1 STAT3 is highly expressed in GB with a high prognostic value.**

(A-B) Expression analysis of STAT3 mRNA in TCGA and CGGA databases: a comparison between GB (n = 163) and non-tumor (n = 207) patient samples (A), and a comparison among different grades of glioma (B). (C) OS curves for GB patients with different STAT3 expression levels in CGGA (*P* < 0.01) database. (D) Multivariate Cox analysis of the ability of STAT3 expression and other clinicopathological variables to predict the OS of GB patients. The results are presented as the hazard ratio (HR). The bar represents the HR values' 95% confidence interval (CI). (E) Time‐dependent ROC curves and AUC values for 1‐, 3‐ and 5‐year OS prediction. (F) KEGG analysis of STAT3 based on the TCGA-GB database.


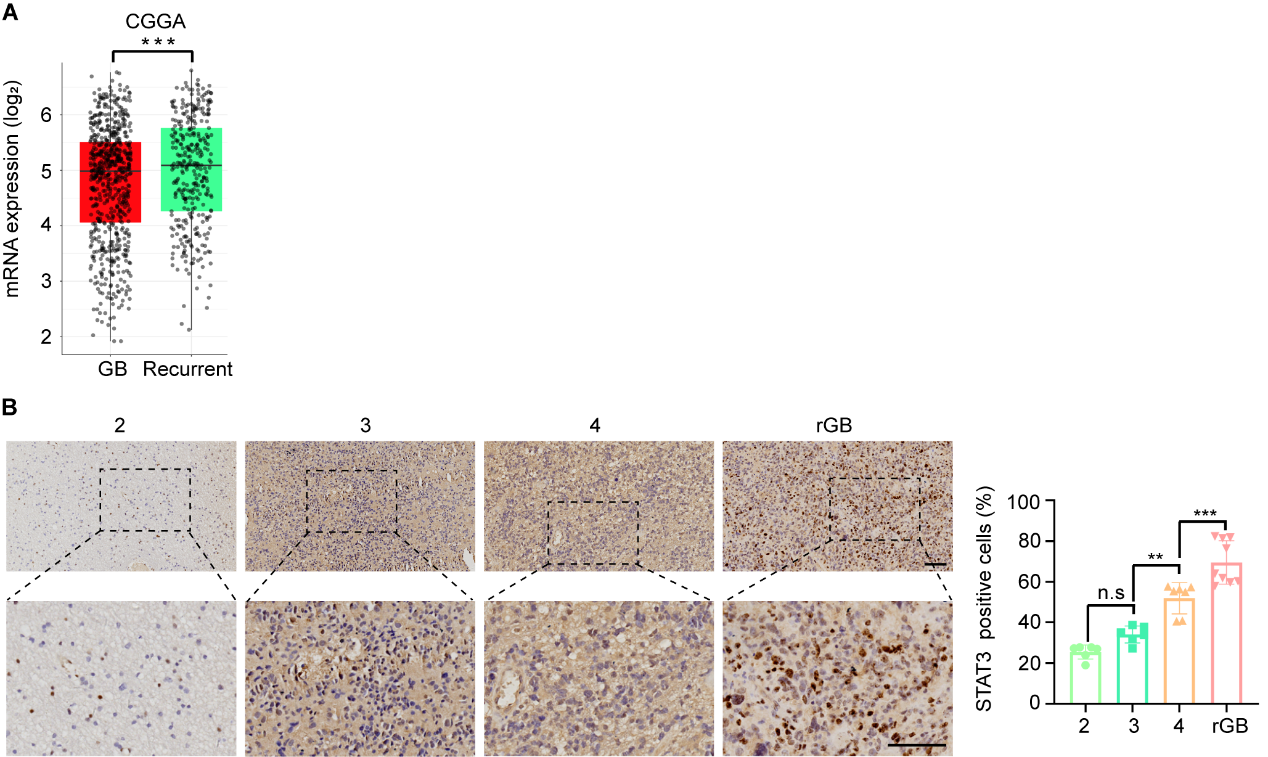


**Fig. S2 The expression of STAT3 was higher in rGB than in GB.**

(A) Comparison of STAT3 mRNA between GB and recurrent GB in CGGA database. (B) IHC analysis was performed for STAT3 in 2, 3, 4 grade, and recurrent GB samples. Scale bar, 50 μm. rGB: recurrent GB. All of the data are presented as the means± S.D. One-way ANOVA was used for multigroup comparisons. n. s = not significant. ** *P* < 0.01 and *** *P* < 0.001.


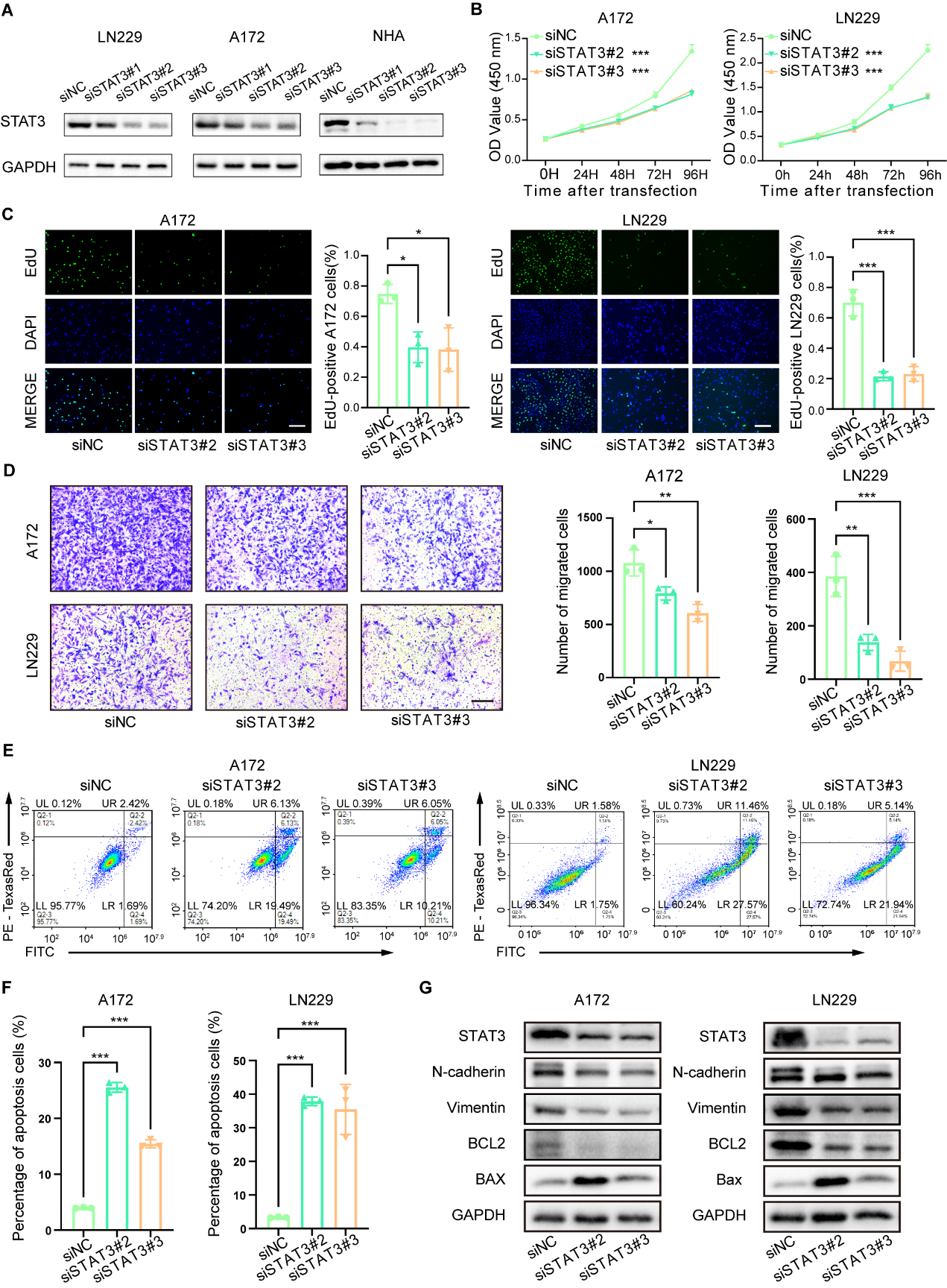


**Fig. S3 STAT3 regulates GB cells’ proliferation, invasion/migration and apoptosis.**

(A) Western blots confirmed the siRNA knockdown efficiency of STAT3 in LN229, A172, and NHAs (siNC, siSTAT3#1, siSTAT3#2, and siSTAT3#3). (B) A Cell Counting Kit-8 assay was employed to measure the viability of A172 and LN229 cells at various time points after seeding. Data points represent the OD450 values. (C) EdU assay was performed on A172 and LN229 cells (blue: all cells; green: proliferating cells) transfected with the specified siRNAs. Data points in histograms indicate the percentage of EdU-positive cells. Scale bar, 50 μm. (D) A trans-well migration assay was conducted for A172 and LN229 cell lines with the indicated treatments. Data points in histograms reflect the number of trans-well cells from A172 and LN229. Scale bar, 50 μm. (E-F) Flow cytometry was used to analyze apoptotic cell percentages in A172 and LN229 with specified treatments. Data points in histograms represent the percentage of apoptotic cells (the sum of the cells in the upper left and lower left quadrants of the flow cytometry results). (G) Western blot analysis examined the expression of STAT3, N-cadherin, Vimentin, BCL2, Bax, and GAPDH in A172 and LN229 cells transfected with the indicated shRNAs. Data are presented as the means ± S.D. (n=3). One-way ANOVA was used for multigroup comparisons. * *P* < 0.05, ** *P* < 0.01 and *** *P* < 0.001.


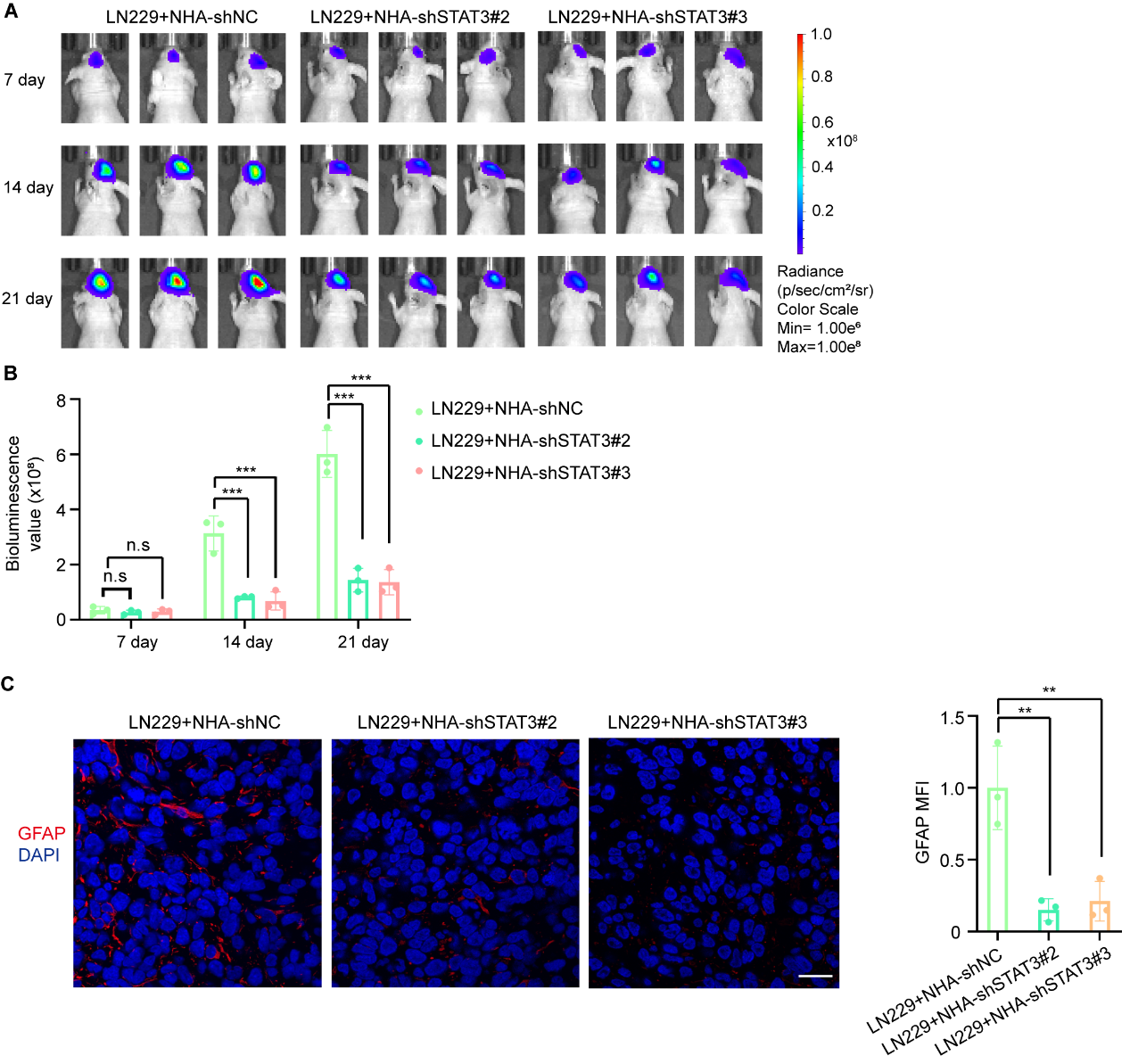


**Fig. S4 Activated astrocytes promoted the progression of GB in vivo**

(A-B) Bioluminescence imaging of mice treated with LN229 together with NHA-shNC, NHA-shSTAT3#2 or NHA-shSTAT3#3. Representative images and statistical analysis at days 7, 14, and 21 are shown. (C) Immunofluorescence staining for GFAP (red) in NHAs in orthotopic xenografts. Cell nuclei were stained with DAPI (blue). The bar plots represent the mean fluorescence intensity (MFI) of GFAP expression. Scale bar, 20 µm. Data are presented as the means ± S.D. (n=3). The statistical comparisons were performed using one-way ANOVA. n. s = not significant, ** *P* < 0.01 and ****P* < 0.001.


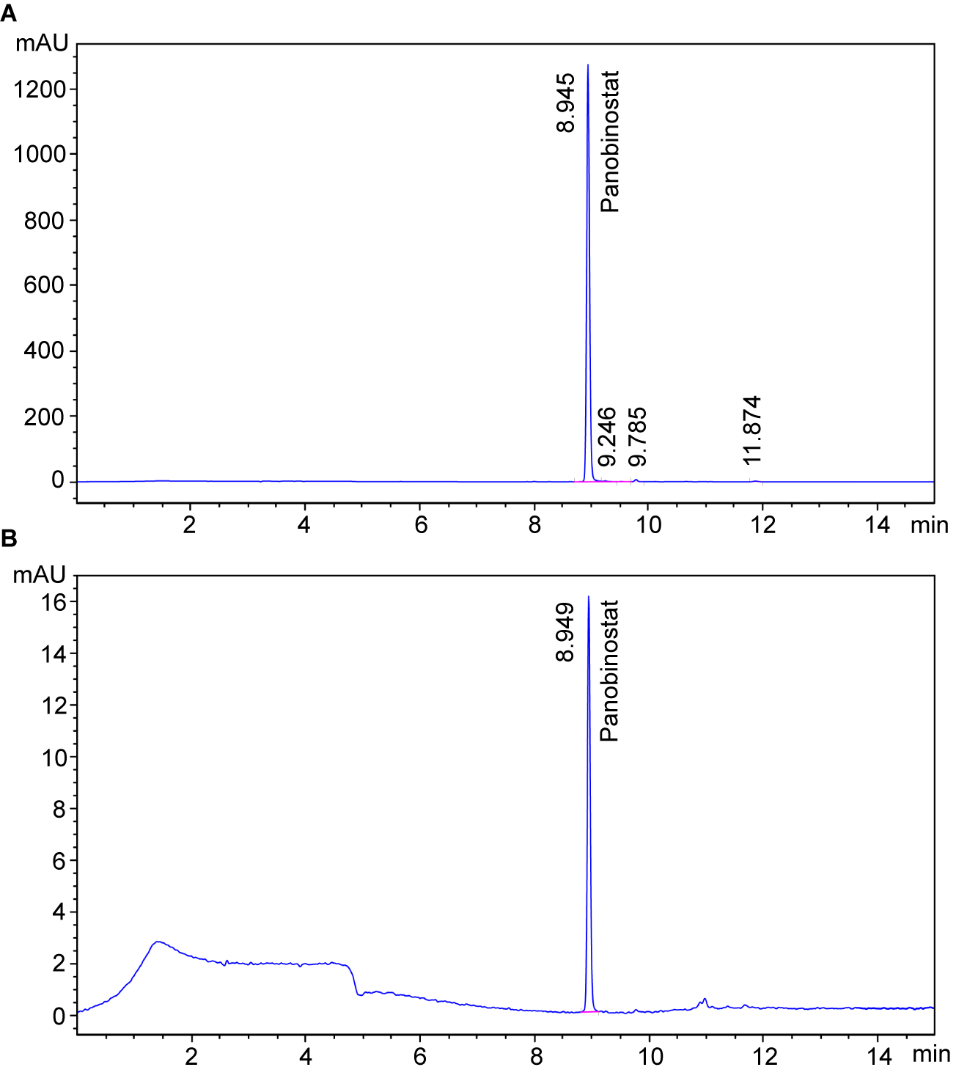


**Fig. S5 Mass spectrometry peak diagram of panobinostat in HPLC experiments.**

The retention times of panobinostat in the standard (A) and test (B) samples are essentially consistent.


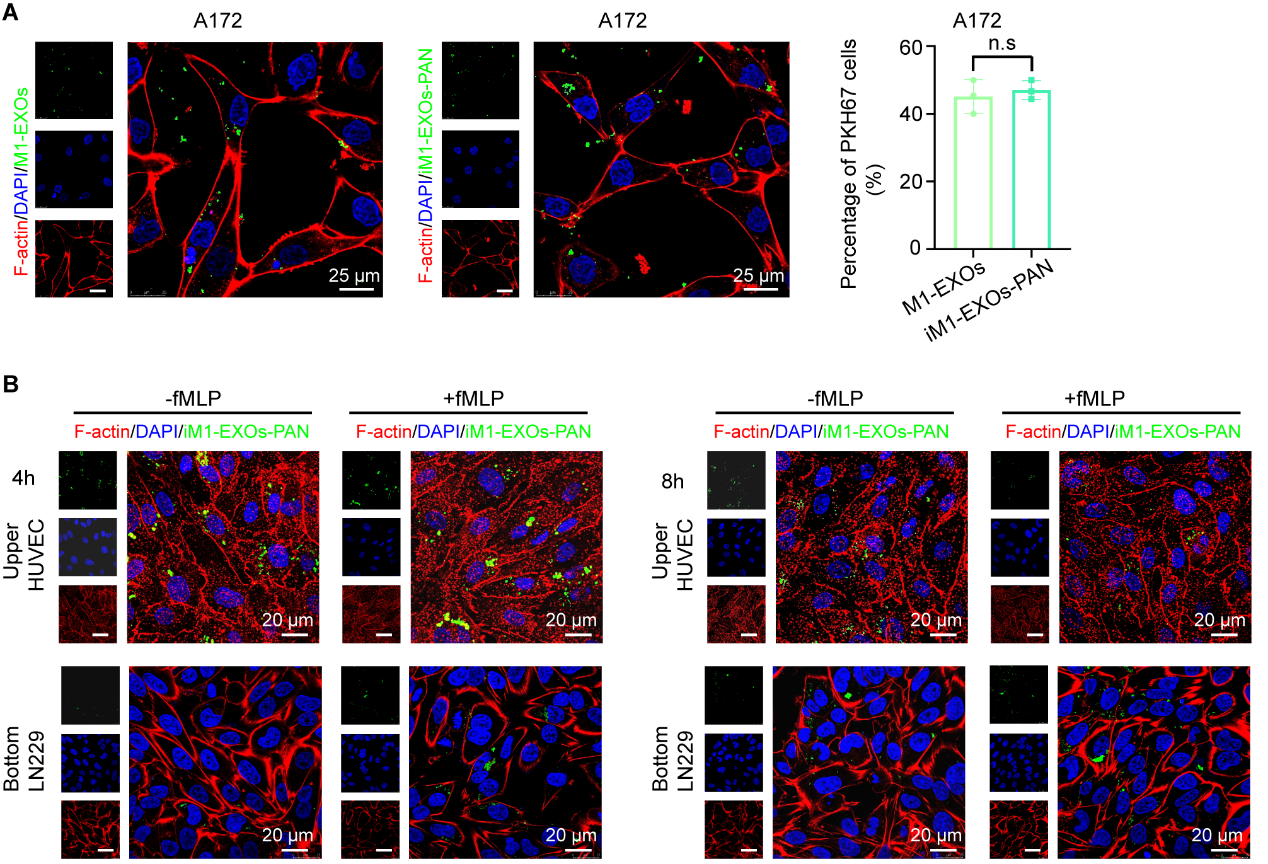


**Fig. S6 Inflammation-directed targeted delivery in vitro.**

(A) Representative confocal images and the quantification of the PKH67‐positive cell ratio showing the uptake of M1-EXOs and iM1-EXOs-PAN by A172 cells. n. s = none-significant. Scale bar, 25 μm. (B) Representative images of HUVECs and LN229 cells taking up the nanoformulation treated with chemotactic peptide (+fMLP, 100 μM) or none (-fMLP) at 4 and 8 hours. Scale bar, 20 μm. Statistical significance was calculated using Student’s *t*-test. n. s = not significant.


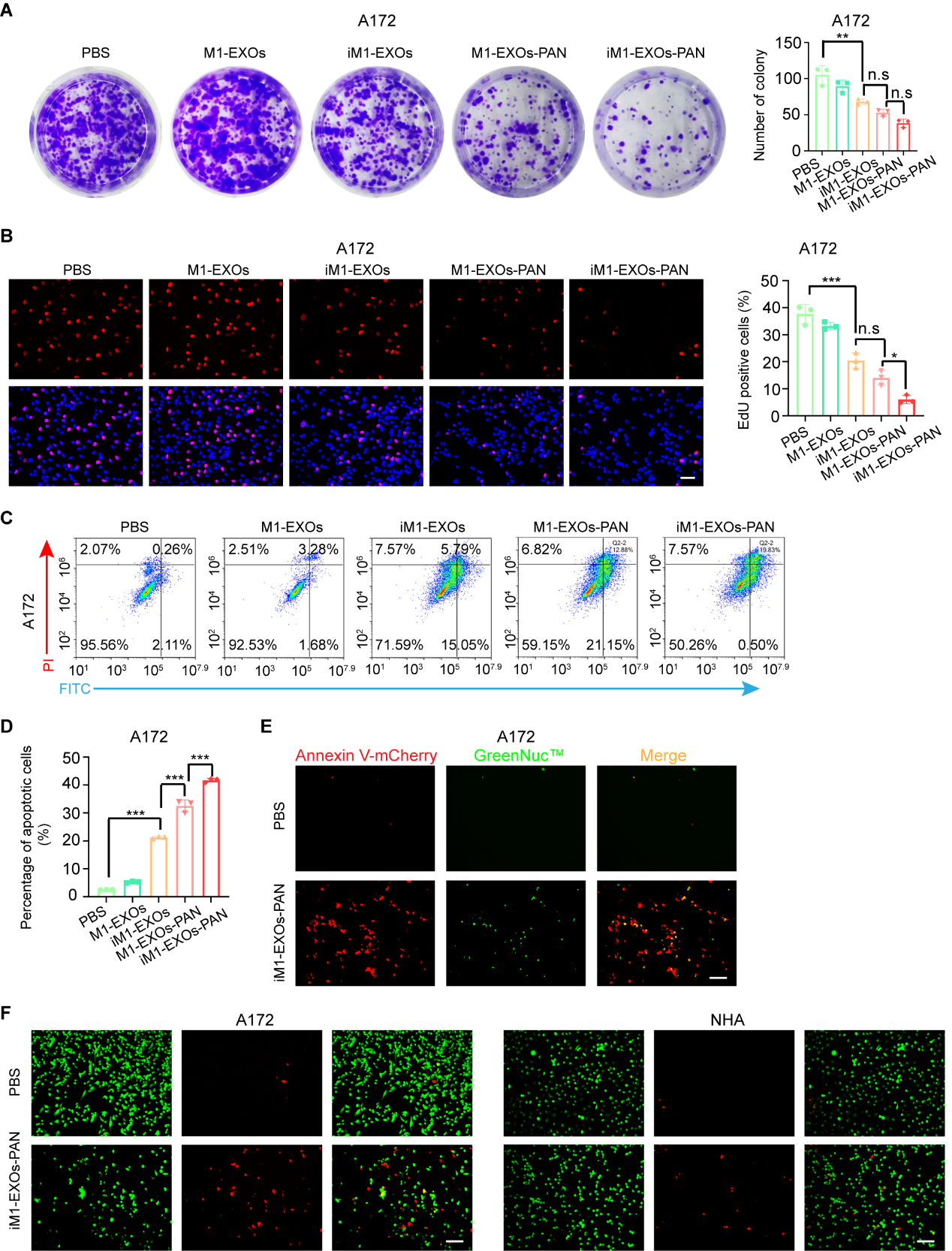


**Fig. S7 iM1-EXOs-PAN represses GB cells proliferation by inducing apoptosis.**

(A) Representative images and statistical analysis for A172 in the colony formation assay with the indicated treatments. (B) Representative images and analysis of EdU-positive cells of A172 with the indicated treatments. Scale bar, 50 μm. (C-D) Apoptosis of A172 was detected by flow cytometry. (E) Representative images of the caspase-3 activity assay in A172 cells (red: cells in apoptosis; green: caspase-3 positive cells). Scale bar, 50 μm. (F) Live-dead staining of A172 and NHA cells (green: live cells; red: dead cells). Scale bar, 50 μm. The data are presented as the means ± S.D. Statistical comparisons were performed using one-way ANOVA. n. s = not significant. **P* < 0.05, ***P* < 0.01 and ****P* < 0.001.


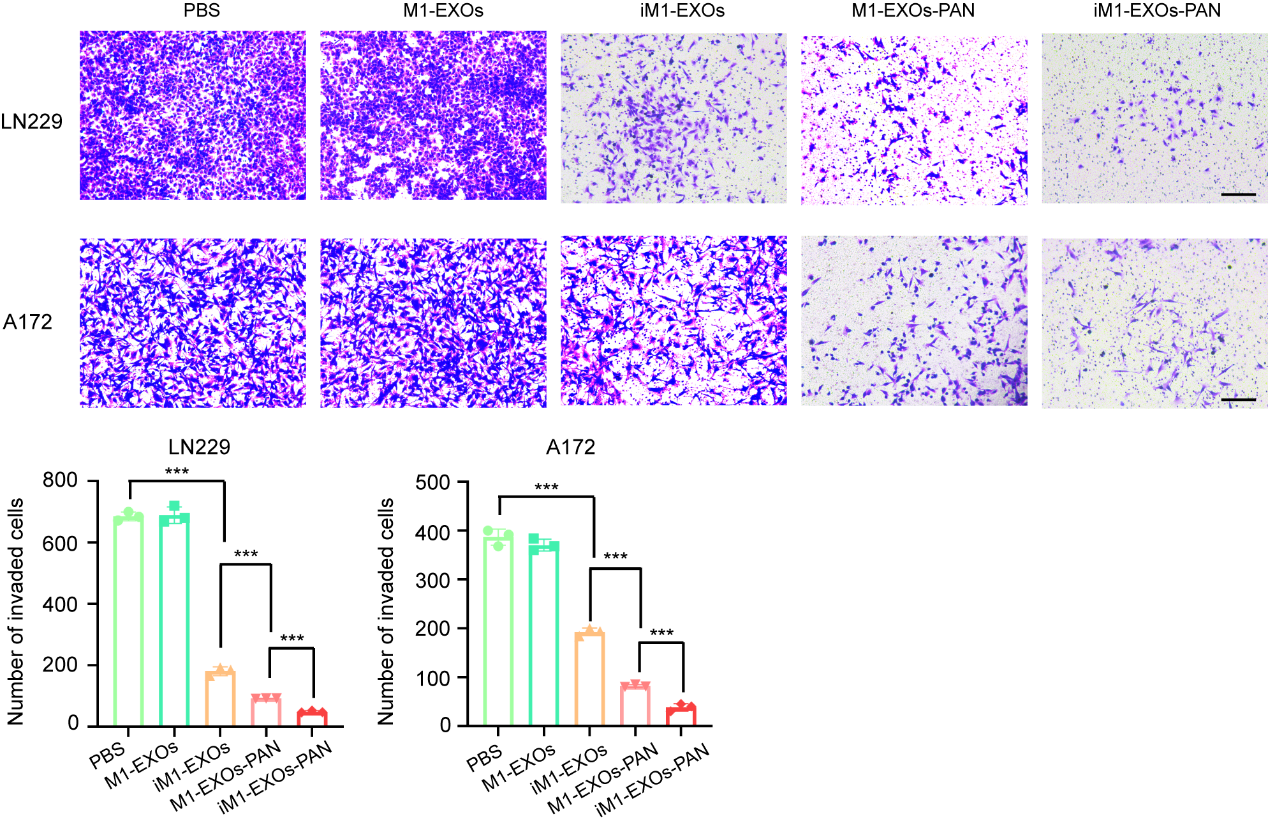


**Fig. S8 iM1-EXOs-PAN inhibits migration and invasion of GB cells.**

Trans-well assays for LN229 and A172 cell lines were conducted with the indicated treatments. Quantification of trans-well cells for LN229 and A172 was performed. Scale bar, 25 μm. The data are presented as the means ± S.D. Statistical comparisons were performed using one-way ANOVA. ****P* < 0.001.


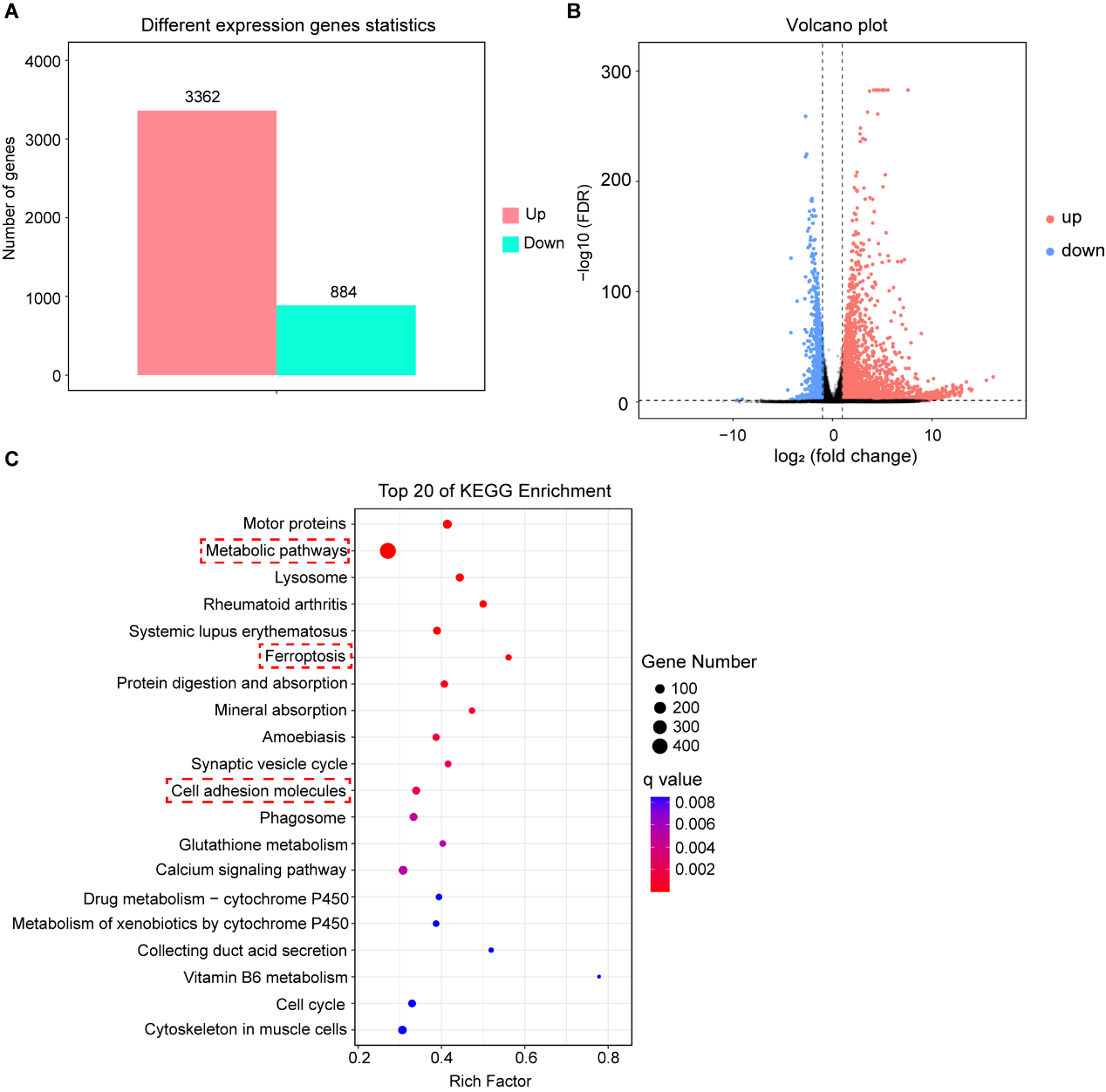


**Fig. S9 RNA-seq demonstrating how iM1-EXOs-PAN affects the transcriptome and essential cell signaling pathways in GB cells.**

(A) Bar chart and (B) volcano plot of different upregulated and downregulated genes in GL261 cells treated with PBS and iM1-EXOs-PAN. (C) KEGG enrichment analysis of the upregulated and downregulated genes in the PBS and iM1-EXOs-PAN treated groups.


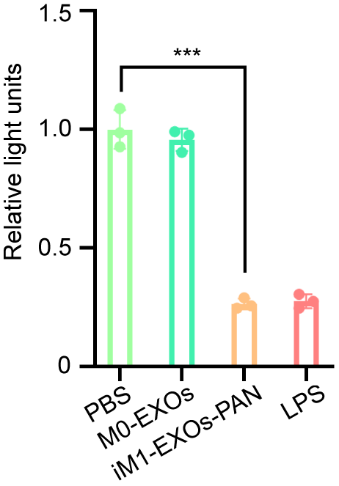


**Fig. S10 Intracellular luciferase activity assay**

In the phagocytosis assay, iM1-EXOs-PAN significantly reduced luciferase activity in GL261 cells. Statistical significance was calculated using Student’s *t*-test. ****P* < 0.001.


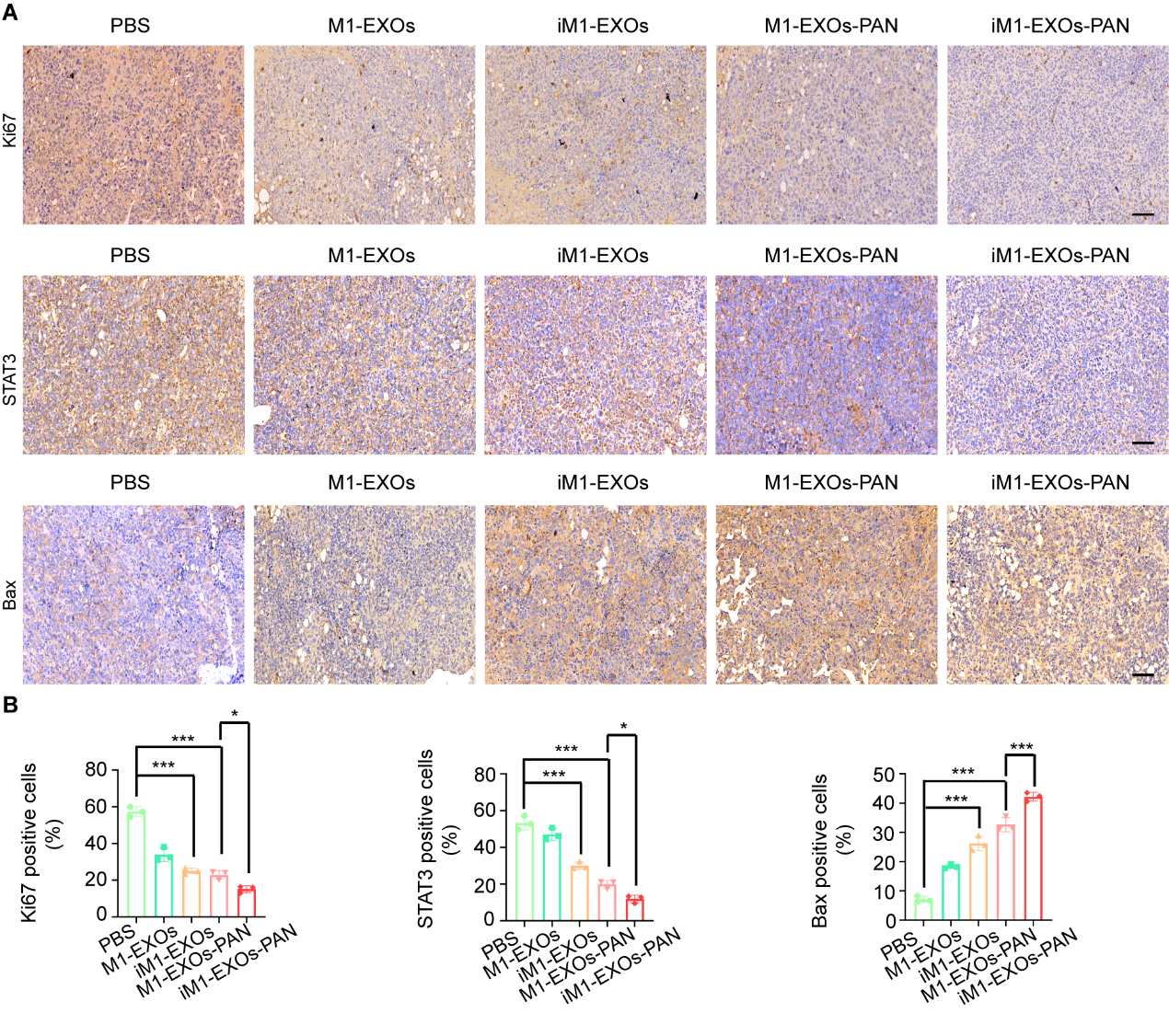


**Fig. S11 iM1-EXOs-PAN significantly inhibits GB recurrence.**

(A) Representative Ki67, STAT3 and Bax immunochemistry images of the xenograft GB tumors after the indicated treatment. Scale bar, 50 μm. (B) Quantification of immunochemistry values. The data are presented as the means ± S.D. Statistical comparisons were performed using one-way ANOVA. **P* < 0.05 and ****P* < 0.001.


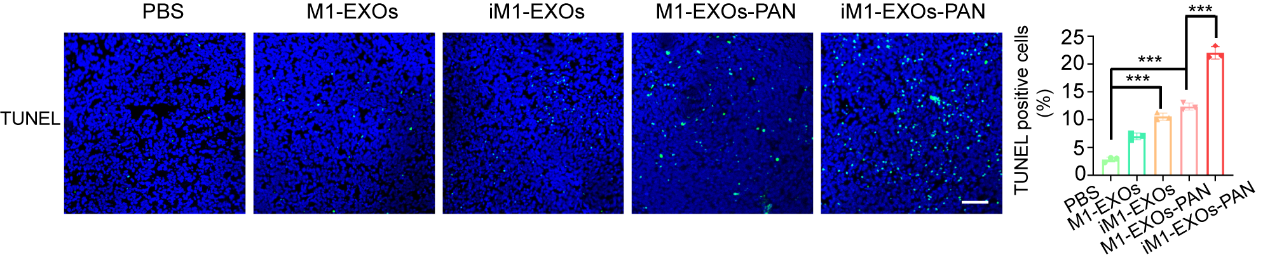


**Fig. S12 iM1-EXOs-PAN significantly inhibits GB recurrence.**

The TUNEL assay was conducted to detect cell apoptosis in recurrent GB sections at the conclusion of the antitumor study. Scale bar, 50 μm. The data are presented as the means ± S.D. Statistical comparisons were performed using one-way ANOVA. ****P* < 0.001.


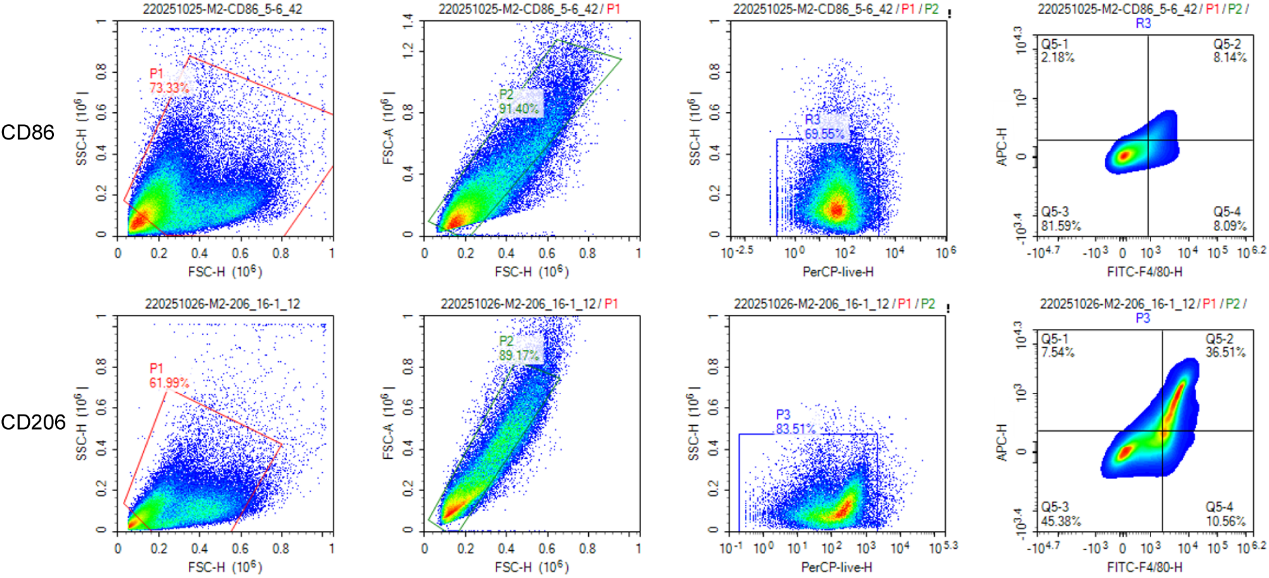


**Fig. S13 Gating strategy plots for the flow cytometry data.**

The upper lane represents the gating method for CD86, while the lower lane shows the gating strategy for CD206.

**Table S1. Primer sequences for qRT-PCR.**

| Gene | Forward (5’-3’) | | Reverse (5’-3’) |
| --- | --- | --- | --- |
| iNOS | CCTGCTTTGTGCGAAGTGTC | | CCCAAACACCAAGCTCATGC |
| FNF-α | | CGGGCAGGTCTACTTTGGAG | ACCCTGAGCCATAATCCCCT |
| IL-10 | | GCTCTTGCACTACCAAAGCC | CTGCTGATCCTCATGCCAGT |
| IL-6 | | CTTCTTGGGACTGATGCTGGT | CTCTGTGAAGTCTCCTCTCCG |
| IL-4 | | GTCCTCACAGCAACGAAGAACAC | CAGGCATCGAAAAGCCCGAA |
| GAPDH | | TGTCTCCTGCGACTTCAACA | GGTGGTCCAGGGTTTCTTACT |
